# The “limbic network,” comprising orbitofrontal and anterior temporal cortex, is part of an extended default network: Evidence from multi-echo fMRI

**DOI:** 10.1162/netn_a_00385

**Published:** 2024-10-01

**Authors:** Manesh Girn, Roni Setton, Gary R. Turner, R. Nathan Spreng

**Affiliations:** Department of Neurology and Neurosurgery, Montreal Neurological Institute, McGill University, Montreal, QC, Canada; Neuroscape, Department of Neurology, University of California San Francisco, San Francisco, CA, USA; Department of Psychology, Harvard University, Cambridge, MA, USA; Department of Psychology, York University, ON, Canada

**Keywords:** Default mode network, Resting-state functional connectivity, Limbic, Multi-echo fMRI, Temporal pole, Ventromedial, Prefrontal

## Abstract

Resting-state functional magnetic resonance imaging (fMRI) investigations have provided a view of the default network (DN) as composed of a specific set of frontal, parietal, and temporal cortical regions. This spatial topography is typically defined with reference to an influential network parcellation scheme that designated the DN as one of seven large-scale networks (Yeo et al., 2011). However, the precise functional organization of the DN is still under debate, with studies arguing for varying subnetwork configurations and the inclusion of subcortical regions. In this vein, the so-called limbic network—defined as a distinct large-scale network comprising the bilateral temporal poles, ventral anterior temporal lobes, and orbitofrontal cortex—is of particular interest. A large multi-modal and multi-species literature on the anatomical, functional, and cognitive properties of these regions suggests a close relationship to the DN. Notably, these regions have poor signal quality with conventional fMRI acquisition, likely obscuring their network affiliation in most studies. Here, we leverage a multi-echo fMRI dataset with high temporal signal-to-noise and whole-brain coverage, including orbitofrontal and anterior temporal regions, to examine the large-scale network resting-state functional connectivity of these regions and assess their associations with the DN. Consistent with our hypotheses, our results support the inclusion of the majority of the orbitofrontal and anterior temporal cortex as part of the DN and reveal significant heterogeneity in their functional connectivity. We observed that left-lateralized regions within the temporal poles and ventral anterior temporal lobes, as well as medial orbitofrontal regions, exhibited the greatest resting-state functional connectivity with the DN, with heterogeneity across DN subnetworks. Overall, our findings suggest that, rather than being a functionally distinct network, the orbitofrontal and anterior temporal regions comprise part of a larger, extended default network.

## Author Summary

The precise functional organization of the default network is still under debate. Limitations in temporal signal-to-noise of functional MRI BOLD signal data may have restricted estimations of the topography of the default network. The “limbic network,” defined as a distinct large-scale network comprising bilateral anterior temporal and orbitofrontal cortex, has been affiliated with the default network in nonhuman animal tractography and task-based fMRI studies in humans. We leverage a multi-echo fMRI dataset with high temporal signal-to-noise and whole-brain coverage to examine the large-scale network resting-state functional connectivity of these regions and assess their associations with the default network. Our results support the inclusion of anterior temporal and orbitofrontal cortex as part of the default network. Overall, our findings suggest that, rather than being a functionally distinct limbic network, the anterior temporal and orbitofrontal regions comprise part of an extended default network.

## INTRODUCTION

Technological and methodological advances in functional magnetic resonance imaging (fMRI) research over the past three decades have afforded an unprecedented ability to characterize the functional organization of the human brain (Raichle, 2009). Chief among these approaches is resting-state functional connectivity (RSFC), which assesses the interregional correlation structure of the brain while an individual is not engaged in an explicit task (Biswal et al., 1995; Buckner et al., 2013). RSFC investigations have revealed that the brain comprises a set of reliable and consistent large-scale networks that interact to mediate perception, cognition, emotion, and behavior (Bassett & Sporns, 2017; Bressler & Menon, 2010; Damoiseaux et al., 2006; Uddin et al., 2019). The rise of RSFC approaches led to the discovery that a set of regions that consistently deactivated during cognitive tasks were strongly positively correlated in the absence of any overt task (Raichle & Snyder, 2007). This set of regions, now referred to as the “default network” (DN), has been the focus of a considerable amount of research, and the DN has now been linked to a variety of complex cognitive processes (Andrews-Hanna et al., 2014; Buckner & DiNicola, 2019; Kleckner et al., 2017; Smallwood et al., 2021).

RSFC studies have characterized the DN as a set of regions spanning the frontal, temporal, and parietal lobes (Buckner et al., 2008; Buckner & DiNicola, 2019; Raichle et al., 2001; Smallwood et al., 2021; Yeo et al., 2011). These studies generally characterize the DN as consisting of dorsal, anterior, and ventral medial prefrontal cortex, posterior cingulate/retrosplenial cortex, inferior parietal lobule, lateral temporal cortex, as well as the parahippocampus and hippocampus. RSFC and meta-analytic task findings have further supported a fractionation of the DN into three subnetworks: (a) the DN_A_, which includes the anteromedial prefrontal cortex and dorsal posterior cingulate cortex; (b) the DN_B_, which includes the dorsomedial prefrontal cortex, anterior inferior parietal lobule/temporoparietal junction, and lateral temporal cortex; and (c) the DN_C_, which includes ventromedial prefrontal cortex, posterior inferior parietal lobule, parahippocampus, and hippocampus (Andrews-Hanna et al., 2010; Yeo et al., 2011).

Despite these advances, the precise organization of the DN is still a matter of debate (Uddin et al., 2019). For example, there is disagreement about whether the DN consists of the three partially dissociable systems described above, or composed of two dissociable systems that are conflated as a result of group-level averaging and the obscuring influence of individual differences in functional neuroanatomy (Andrews-Hanna et al., 2010; Braga & Buckner, 2017; Buckner & DiNicola, 2019; DiNicola et al., 2020; Yeo et al., 2011). A line of work has also pushed back against the prevalence of corticocentric views of the DN and has provided evidence for the inclusion of subcortical regions as a core part of this network (Alves et al., 2019; Buckner et al., 2011; Bzdok et al., 2013; Choi et al., 2012; Cunningham et al., 2017; Kleckner et al., 2017). An additional area of interest with regard to the functional organization of the DN is the so-called limbic network (LIM; Yeo et al., 2011) and its potential inclusion as part of the DN (see Uddin et al., 2019).

The LIM was defined by the highly cited Yeo et al. (2011) network parcellation scheme as a distinct network that is composed of two subnetworks: LIM_A_, which encompasses the temporal pole (TP) and adjacent regions of the ventral anterior temporal lobe (vATL); and LIM_B_, which corresponds to orbitofrontal cortex (OFC). The reliability of these regions forming a distinct network, however, is unclear given that they have among the least reliable signal in conventional fMRI acquisition protocols. This is due to their close proximity to nasal airways and consequent vulnerability to susceptibility-related signal loss (Ojemann et al., 1997). Indeed, the Yeo et al. (2011) study featured poor temporal signal-to-noise ratio (TSNR ∼40) in most LIM regions, suggesting that the grouping of these regions into a distinct network may, at least partially, be driven by their shared property of poor signal.

Findings from tract-tracing work in nonhuman primates, as well as RSFC and task-based fMRI investigations where modestly reliable signal is present, suggest that regions within LIM—that is, spanning the TP, vATL, and OFC—may be construed as extensions of the DN. Tract-tracing in nonhuman primates has found that the TP and vATL exhibit extensive anatomical connections to numerous cortical and subcortical regions, many of which correspond to homologs of human DN regions (Barbas et al., 1999; Kondo et al., 2003; Moran et al., 1987; Saleem et al., 2008). Putative DN regions found to exhibit anatomical connectivity with subregions of the TP and vATL in macaque monkeys include the anteromedial and ventromedial prefrontal cortex (Barbas et al., 1999; Kondo et al., 2003), superior and inferior temporal gyrus (Kondo et al., 2003; Moran et al., 1987; Saleem et al., 2008), as well as the parahippocampal cortex and hippocampus (Moran et al., 1987; Muñoz & Insausti, 2005; Saleem et al., 2008). Similarly, the OFC has also been found to exhibit anatomical connections to much of the brain, including regions corresponding to the DN. Putative DN regions found to exhibit anatomical connectivity with the OFC in macaque monkeys include the anteromedial and ventromedial prefrontal cortex (Carmichael & Price, 1996; Price, 2006; Saleem et al., 2008); anterior, mid, and posterior cingulate gyrus (Carmichael & Price, 1995, 1996); inferior frontal gyrus (Carmichael & Price, 1996); superior temporal gyrus (Carmichael & Price, 1995; Kondo et al., 2003; Saleem et al., 2008); as well as the parahippocampal cortex and hippocampus (Carmichael & Price, 1995; Kondo et al., 2005).

Consistent with these findings, RSFC investigations with modest but reliable signal quality (TSNR > 50) in the TP, vATL, and OFC have found evidence for strong RSFC between each limbic network region and the DN. With regard to the TP, one RSFC-based parcellation study found that two anterolateral subdivisions of the left TP—spanning the anterior tip and the middle and inferior temporal gyri, and collectively comprising ∼50% of total TP surface area—strongly recapitulated the full breadth of the DN (Pascual et al., 2015). This RSFC map also included the OFC—suggesting the presence of a combined DN-LIM network (Pascual et al., 2015). Another study parcellated the TP based on anatomical connectivity estimated by diffusion tensor imaging (DTI) and similarly found a bilateral anterolateral subregion that exhibited an RSFC pattern strongly resembling the DN (Fan et al., 2014). Additional studies have also supported the presence of RSFC between TP subregions and the DN (Andrews-Hanna et al., 2010; Jackson et al., 2016; Simmons et al., 2010). Similarly, strong RSFC between the vATL, particularly in the left hemisphere, and regions overlapping with the DN have also been found (Jackson et al., 2016, 2018; Persichetti et al., 2021; Simmons et al., 2010). With regard to the OFC, a RSFC-based parcellation of this region revealed that medial subregions of the OFC in particular were significantly correlated with the medial prefrontal cortex (mPFC) and posterior cingulate cortex (PCC)—two core regions of the DN (Kahnt et al., 2012; see also Kleckner et al., 2017). This finding was consistent with earlier RSFC investigations that, in addition to the mPFC and PCC, also revealed RSFC between the OFC and medial temporal regions such as the parahippocampus and hippocampus (Andrews-Hanna et al., 2010; Vincent et al., 2006). A more recent study assessed OFC RSFC in 654 individuals and again found that multiple subregions—including the medial extent of OFC along the ventral frontal lobes—exhibited significant RSFC with the majority of the DN (Du et al., 2020).

Task-based fMRI investigations provide further evidence for strong linkages between each of the TP, vATL, and OFC, and the DN. A notable example is the common recruitment of the TP, vATL, and OFC alongside the DN in investigations of complex cognitive processes, including social cognition, spontaneous thought, episodic memory recollection and prospection, and self-referential processing (Andrews-Hanna et al., 2014; Dixon et al., 2017; Fox et al., 2018; Frith & Frith, 2007; Spreng et al., 2009). Among these, the coactivation of the TP with DN_B_ for social cognitive processes is a particularly consistent and robust finding (Andrews-Hanna et al., 2014; Frith & Frith, 2007; Simmons et al., 2010; Spreng et al., 2009). The TP and vATL are also central regions within a distributed conceptual processing/semantic memory network that highly overlaps with DN_B_ (Binder et al., 2009; Ralph et al., 2017). Further examples with respect to the OFC come from two meta-analytic connectivity modeling investigations of this region based on the Brain Map database (Chase et al., 2020; Zald et al., 2014). These studies revealed—directly in line with RSFC studies—that subregions of the OFC consistently exhibit significant task-based coactivation with the mPFC, PCC, and medial temporal lobes (parahippocampus and hippocampus) across a variety of cognitive tasks (Chase et al., 2020; Zald et al., 2014).

**Figure 1.**
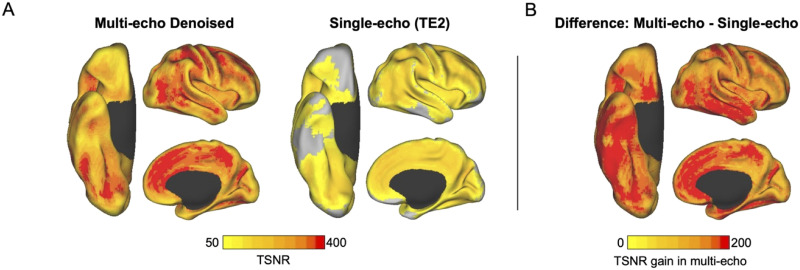
Gain in temporal signal-to-noise ratio with multi-echo data evident in lateral temporal and orbitofrontal cortices. Spatial maps of the temporal signal-to-noise ratio were calculated by participant in MNI space and averaged across the full sample. (A) Multi-echo denoised data (left) compared with minimally preprocessed single-echo data (right). Images acquired from the second echo were used as a proxy for single-echo data as the echo time is comparable to that in single-echo acquisitions. (B) The difference between maps, calculated as multi-echo minus single-echo temporal signal-to-noise, illustrates the relative gain in BOLD signal sensitivity with multi-echo acquisitions. This difference is especially prominent (darker red) in lateral temporal and orbitofrontal cortices. TE2 = echo time 2; TSNR = temporal signal-to-noise ratio.

Collectively, therefore, there is a strong body of evidence suggesting that the TP/vATL and OFC—or subregions thereof—may be regarded as part of the DN. However, this has yet to be evaluated in a targeted investigation. We leverage an open-access multi-echo fMRI dataset (Spreng et al., 2022) that affords exceptional signal quality in LIM regions, thereby overcoming limitations concerning poor LIM TSNR in past work. Briefly, the signal of a given brain region at a given echo time (TE) is based on the transverse relaxation time (T2*) of brain tissue in that region. Brain regions vary in T2*, and therefore a given TE will result in higher TSNR in some regions and lower TSNR in others. In typical fMRI acquisitions, data are collected at a single TE with the goal of maximizing signal quality across the entire brain; however, this approach results in notable trade-offs, such as poor signal in the TP/vATL and OFC. Critically, the multi-echo acquisition used here collected data at three TEs, including a shorter TE that exhibits significantly less signal dropout in regions vulnerable to susceptibility distortions (Kundu et al., 2017). We optimally combined each TE time series in a manner that optimized for signal quality on a voxel-wise level, thereby mitigating signal dropout and significantly boosting whole-brain TSNR (see Figure 1). Moreover, the use of multi-echo data enabled the use of multi-echo independent component analysis (ME-ICA) denoising, a biophysically based noise-removal technique that separates BOLD from non-BOLD signal based on TE dependence. This principled denoising technique has been found effective in removing motion and physiological artifacts in resting-state fMRI data (Kundu et al., 2012, 2013; Lynch et al., 2020; Setton et al., 2023), including distant-dependent motion effects (Power et al., 2018; Spreng et al., 2019).

Combining multi-echo resting-state fMRI data and ME-ICA denoising affords the ability to map the RSFC and network organization of LIM_A_ (TP/vATL) and LIM_B_ (OFC). Leveraging these data, we sought to (a) determine whether data-driven network parcellation approaches assign LIM regions to the same network as DN regions, (b) assess the whole-brain RSFC of LIM_A_ and LIM_B_ subnetworks, and (c) assess the presence of functional heterogeneity within LIM_A_ and LIM_B_ via data-driven clustering. We hypothesize that (a) data-driven network parcellation approaches will assign the majority of LIM regions to the DN, (b) data-driven clustering will delineate functionally heterogenous subregions within the LIM, and (c) left-lateralized LIM_A_ and medial LIM_B_ regions will exhibit the greatest RSFC with the DN, given the former’s overlap with the DN-overlapping semantic system (Binder et al., 2009; Ralph et al., 2017), and the latter’s previously documented RSFC with DN regions (Du et al., 2020; Kahnt et al., 2012).

## METHODS

### Participants

A total of 154 young adults (mean age 22.29, *SD*: 3.12; range: 18–34 years; 86 women) participated in the current study. All participants were healthy and had no history of psychiatric, neurological, or other medical illness that could compromise cognitive function.

### Neuroimaging Data Acquisition

All imaging data were acquired on a 3T GE Discovery MR750 scanner (General Electric, Milwaukee, United States) with a 32-channel receive-only phased-array head coil at the Cornell MRI facility in Ithaca. High-resolution structural images were acquired during one 5-m, 25-s run using a T1-weighted (T1w) volumetric MRI magnetization prepared rapid gradient echo (MPRAGE) sequence (repetition time [TR] = 2,530 ms; echo time [TE] = 3.4 ms; inversion time [TI] = 1,100 ms; flip angle [FA] = 7°; bandwidth = 195 Hz/pixel; 1.0 mm isotropic voxels, 176 slices). Structural scans were acquired with 2× acceleration with sensitivity encoding.

Participants completed two 10-min, 6-s resting-state multi-echo BOLD functional scans with eyes open, blinking and breathing normally in the dimly lit scanner bay. These scans were acquired using a multi-echo echo planar imaging (ME-EPI) sequence with online reconstruction (TR = 3,000 ms; TEs = 13.7, 30, 47 ms; FA = 83°; matrix size = 72 × 72; field of view [FOV] = 210 mm; 46 axial slices; 3.0 mm isotropic voxels). Resting-state functional scans were acquired with 2.5× acceleration with sensitivity encoding and were acquired prior to engagement in any cognitive task.

### Neuroimaging Preprocessing and Denoising

Resting-state fMRI data were collected at three echo times (TEs), as afforded by the multi-echo fMRI acquisition. Time series data at each TE were first minimally preprocessed: the first four volumes were discarded, images were computed for de-obliquing, motion correction, and anatomical-functional coregistration, and volumes were brought into spatial alignment across TEs.

Given interregional differences in T2* relaxation rates, volumes collected at each TE result in differential signal quality across regions. To exploit this, the resting-state fMRI data were averaged across TEs in a manner that was optimally weighted to maximize the temporal signal-to-noise ratio (TSNR) of each voxel. This significantly improves whole-brain TSNR and, critically, attenuates signal dropout in typically problematic regions along the ventral-anterior surface of the brain (i.e., orbitofrontal cortex and the temporal pole; Kundu et al., 2012, 2013; Lynch et al., 2020).

In addition, a multi-echo acquisition facilitates the biophysically based removal of noise components from resting-state fMRI datasets (Kundu et al., 2012, 2013). This is because collecting data at multiple TEs allows the direct measurement of TE-dependent variability in the signal. The denoising method presently employed—multi-echo independent component analysis (ME-ICA)—exploits this information to distinguish BOLD signal from non-BOLD noise (Kundu et al., 2012). TE-dependent variability of the signal can be fit to models of changes in T2* (i.e., the transverse relaxation rate; the basis for the BOLD contrast) or changes in baseline signal (S_0_) that are the product of scanner artifacts, motion, and other sources of noise (Kundu et al., 2012). By comparing the relative goodness of fit of TE dependence with each of these models, one can separate BOLD signal from non-BOLD noise. Past work has supported the effectiveness of this technique in denoising BOLD signal of motion and physiological artifacts in resting-state fMRI (Kundu et al., 2012, 2013; Lynch et al., 2020; Setton et al., 2023). Importantly, ME-ICA denoising has been found to remove distant dependent motion effects from RSFC data (Power et al., 2018; Spreng et al., 2019). ME-ICA outputs include (a) spatial maps consisting of the BOLD-like components, (b) reconstructed time series based on back-projecting the BOLD-like components only, and (c) the BOLD-like component coefficient sets.

Quality assessments were performed on the reconstructed time series (ME-ICA Output b) in native space to identify and exclude participants with unsuccessful coregistration, residual noise (framewise displacement > 0.50 mm coupled with denoised time series showing DVARS > 1), temporal signal-to-noise ratio < 50, or fewer than 10 retained BOLD-like components. The denoised BOLD component coefficient sets (ME-ICA Output c) in native space, optimized for functional connectivity analyses (Kundu et al., 2013), were used in subsequent steps. We refer to these BOLD component coefficient sets as multi-echo functional connectivity (MEFC) data. Additional measures were taken to account for variation in the number of independent components from ME-ICA once connectivity matrices were estimated, as detailed below.

ME-ICA processing was run with ME-ICA version 3.2 beta (https://github.com/ME-ICA/; Kundu et al., 2012, 2013). Anatomical images were first skull stripped using the default parameters in FSL BET. ME-ICA processing was then run with the following options: -e 13.6, 29.79, 46.59; -b 12; –no_skullstrip; –space = Qwarp_meanE+tlrc. Here, the Qwarp_meanE+tlrc file represented a site-specific MNI-space template (available here: https://zenodo.org/record/3575255). This template was created in AFNI using @toMNI_Qwarpar. MNI-space ME-ICA BOLD coefficient time series were resampled to 2 mm isotropic. Time series were not smoothed, given that the parcel-wise time series extraction approach applied here represents a de facto form of smoothing (i.e., by averaging across spatially contiguous voxels).

### Signal Quality

In order to assess the whole-brain signal quality of the ME-ICA processed images, TSNR was calculated for each voxel as the mean signal intensity across its time series, divided by its standard deviation. Derived TSNR spatial maps were averaged across all subjects and thresholded at 50 (Figure 1). To illustrate the boost in TSNR relative to single-echo data, TSNR maps were calculated on minimally preprocessed images (i.e., de-obliqued, motion-corrected, and warped to MNI space) from the second echo for each participant and averaged across all subjects. Difference maps were also created by subtracting the average single-echo map from the average multi-echo map (as in Steel et al., 2022; Figure 1). The results indicate strong whole-brain coverage, including within limbic areas that typically exhibit signal dropout (i.e., orbitofrontal cortex and the temporal pole), consistent with prior reports of ME-ICA (e.g., DuPre et al., 2016; Setton et al., 2023).

### Neuroimaging Data Analysis

#### Resting-state functional connectivity.

The MEFC denoised resting-state fMRI data (see the Neuroimaging Preprocessing and Denoising section above) were parcellated into 1,032 regions as follows: 1,000 cortical regions following Schaefer et al. (2018) and 7 subcortical regions (hippocampus, amygdala, nucleus accumbens, globus pallidus, putamen, caudate as per Tian et al., 2020, and the basal forebrain as per the SPM Anatomy Toolbox thresholded at 50% probability). RSFC was computed as the product-moment correlation coefficient between all parcels, resulting in a 1,032 × 1,032 RSFC matrix for each subject. Given our use of MEFC data, RSFC was calculated as the correlation of the ME-ICA coefficients across parcels (parcel × coefficient vectors), rather than a correlation across BOLD signal time series (parcel × time point vectors), as is typically done (see Kundu et al., 2013). The Fisher’s r-to-z transformation was applied to normalize the distribution of correlation values and, importantly, account for variation in MEFC data degrees of freedom (as quantified by the number of BOLD ME-ICA components), across individuals (Kundu et al., 2013):

$$Z=\arctan\left(R\right)*df-3,$$

where *R* is the product–moment correlation value and *df* is the number of BOLD ME-ICA components.

#### Modularity.

Modularity analyses were applied to obtain data-driven region-to-network assignments. Modularity is a measure derived from the subfield of mathematics referred to as graph theory. The application of graph theory to neuroimaging data formalizes the brain as a network of nodes (e.g., brain regions) that are connected by edges/links (e.g., functional correlations, white matter pathways; Bassett & Sporns, 2017; Rubinov & Sporns, 2010). This formalization enables the quantification of topological properties associated with the brain’s graph (i.e., network) structure. Modularity analyses provide an assessment of the decomposability of a given network (in this case, whole-brain functional connectivity) into distinct modules and assigns each node (i.e., region/parcel) of the network into a particular network.

Two modularity algorithms were applied: the Louvain algorithm and the Infomap algorithm. The Louvain algorithm (Blondel et al., 2008) was implemented with the Network Community Toolbox (https://commdetect.weebly.com/). This algorithm finds modular partitions of the graph (synonymous with network) that optimize the modularity value, *Q*, by grouping nodes into nonoverlapping (sub)networks that maximize intramodular and minimize intermodular connections (Newman, 2004). The modularity value *Q* for a given modular partition is computed as follows:

$$Q=\frac{1}{l}\sum_{i,j\in N}\left[w_{ij}-\frac{k_{i}k_{j}}{l}\right]\delta_{m_{i}mj},$$

where *w* is the edge weight (i.e., functional connectivity value) between nodes *i* and *j*, *l*^w^ is the sum of all weights in the graph, *k*_i_ is the weighted degree (edge weight summed across all edges) of node *i*, and *m*_i_ is a module containing node *i*. The term *δ*_*m*_*i*_*m*_*j*__ equals 1 if nodes *i* and *j* belong to the same module, and 0 otherwise. The *Q* value for a given partition therefore quantifies the strength of within-module edges relative to the strength of between-module edges, or, in other words, the extent to which distinct modules can be delineated in the data. This algorithm has a single free parameter, gamma (*γ*), which controls how many modules will be detected. Modularity was computed with three gamma values: 1 (the default), 1.25, and 1.5. The Louvain algorithm was run on the group-level mean RSFC matrix, using the Yeo 7-network assignments as the initial conditions. These initial assignments were used because our goal was to identify whether limbic regions remain assigned to a distinct “limbic network,” or get assigned to one or more of the other large-scale networks.

The group-level mean RSFC matrix was also used as input to the Infomap algorithm, as implemented at https://www.mapequation.org/infomap/ (Rosvall & Bergstrom, 2007). Rather than applying modularity maximization, Infomap estimates communities (i.e., networks/modules) based on the probabilistic flow of information through a network. In particular, this algorithm seeks to minimize the description length of random walks on the network, conceptualized in the context of a map equation. This map equation quantifies the average code length per step of a random walk and delineates communities as groups of nodes among which the random walk is more likely to remain confined, indicating strong internal connectivity. In practice, the algorithm iteratively partitions the network into modules by optimizing the map equation. This optimization is achieved through a heuristic search process, which involves exploring various potential community structures to find the one that most effectively compresses the description of the random walk.

#### Clustering.

Clustering analyses were applied as a data-driven assessment of subnetwork organization within the limbic network. To prepare the data for clustering, eta squared similarity was computed on matrices corresponding to RSFC between limbic regions (61 regions as defined based on the Schaefer-Yeo 17-network, 1,000-region parcellation) and the rest of the brain (i.e., on subject-wise 61 × 1,032 RSFC matrices). This yielded a 61 × 61 RSFC similarity matrix per subject, wherein each value represents the similarity between two given limbic regions in their whole-brain RSFC profile.

Clustering was performed on the group-level mean similarity matrix using Ward’s agglomerative clustering. This clustering algorithm was chosen as past RSFC research has found that it produces results that are more accurate and reproducible than other popular algorithms (i.e., k-means clustering or spectral clustering; Thirion et al., 2014). In addition, it does not require the a priori specification of number of clusters. Ward’s clustering is an unsupervised algorithm that iteratively merges clusters in the data while seeking to minimize the “error sum of squares,” which is computed as the sum of squares of the deviations from the cluster centroid. At initialization, all vectors (in this case, 1 × 61 vectors representing a given LIM parcel’s similarity to all other LIM parcels) are their own cluster, and the algorithm stops after the further merging of clusters does not reduce the error sum of squares.

#### RSFC maps.

We refer to the whole-brain interregional RSFC of a given parcel, subnetwork, or cluster as an “RSFC map.” For a given parcel, this corresponds to a row of the whole-brain RSFC matrix (i.e., a 1 × 1,032 vector). For a given subnetwork or cluster, this corresponds to the mean across the parcels comprising that subnetwork or cluster. Mass univariate one-sample *t* tests were computed across subjects for all RSFC maps of interest (*p* < 0.01 Bonferroni; 1,032 comparisons). To highlight the unique topography of RSFC maps of interest, maps were further thresholded to include only the (absolute) top 10% of connections. Mass univariate paired-sample *t* tests were computed to assess pair-wise differences between RSFC maps of interest (*p* < 0.01 Bonferroni; 1,032 comparisons). Contrasts were also computed on the mean values for subcortical regions of interest, as well as at the network-wise level for the 17 networks defined by the Yeo et al. (2011) parcellation.

Large-scale networks include visual network A (VIS_A_), visual network B (VIS_B_), somatomotor network A (SMN_A_), somatomotor network B (SMN_B_), dorsal attention network A (DAN_A_), dorsal attention network B (DAN_B_), salience network A (SAL_A_), salience network B (SAL_B_), frontoparietal network A (FPN_A_), frontoparietal network B (FPN_B_), frontoparietal network C (FPN_C_), default network A (DN_A_), default network B (DN_B_), default network C (DN_C_), and temporal parietal network (TPar), in addition to LIM_A_ and LIM_B_.

Subcortical regions include the hippocampus (HIP), amygdala (AMY), nucleus accumbens (NAC), globus pallidus (GP), putamen (PUT), caudate (CAU), and basal forebrain (BF).

## RESULTS

### Modularity Results

In order to assess, in a data-driven manner, whether limbic regions comprise a distinct large-scale network or may be more accurately construed as a part of the default network or other large-scale network, we applied two modularity algorithms (see the Modularity section above) to the group-level mean whole-brain RSFC data. These algorithms assign each parcel to a given module (broadly synonymous with network or cluster) based on their RSFC profile.

**Figure 2.**
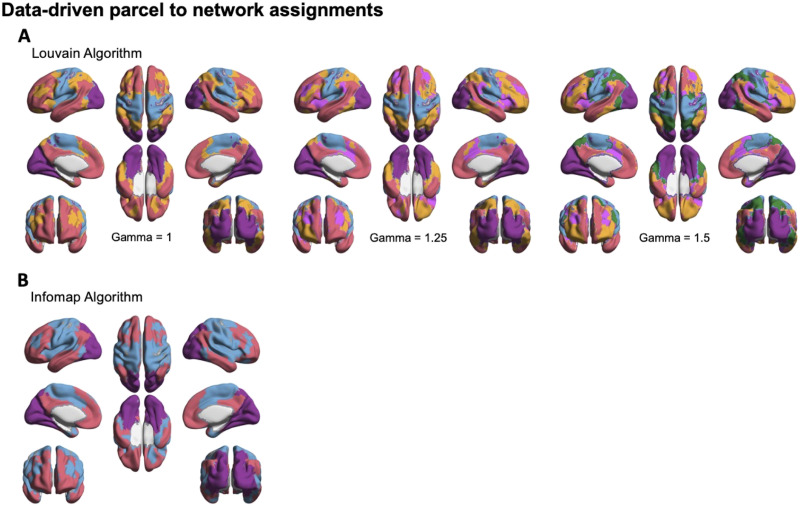
Data-driven parcel network assignments. (A) Data-driven network assignments based on the Louvain modularity algorithm, at three values of the gamma resolution parameter. (B) Data-driven network assignments based on the Infomap modularity algorithm. Networks are colored according to their putative corresponding large-scale network according to the Yeo 7-network parcellation scheme (Yeo et al., 2011).

Modularity was first computed using the Louvain modularity algorithm (Figure 2A). This algorithm requires specification of a gamma parameter that controls the resolution of the output (i.e., number of modules). Three values for the gamma resolution parameter were examined: 1 (the default), 1.25, and 1.5. Results with gamma = 1 parcellated the brain into putative visual (purple), somatomotor (blue), frontoparietal/executive (orange), and default networks (light red). Results with gamma = 1.25 mirrored the results with gamma = 1, with the further differentiation of the putative salience network (magenta). Finally, results with gamma = 1.5 mirrored the results with gamma = 1.25, with the further differentiation of the putative dorsal attention network (green). Critically—consistent with our hypothesis—LIM parcels were assigned to the putative DN across resolutions, with the single exception of parcels within the right medial temporal pole for gamma = 1 and 1.5. Results were consistent across runs with minor differences (Supplementary Figure 1 in the Supporting Information). Louvain modularity results were also computed with gamma = 2 and 2.5 (Supplementary Figure 2). At these higher resolutions, the brain was separated into 13–14 networks (gamma = 2) or 19–20 networks (gamma = 2.25). Consistent with the parcel-wise RSFC maps and clustering results, medial OFC and anterolateral aspects of the vATL remain assigned to the DN at these resolutions. Other parcels—also consistent with the RSFC mapping results—were assigned to a combination of the frontoparietal control network (mostly lateral prefrontal cortex), somatomotor network, or temporoparietal network.

To assess the dependence of these results on the modularity algorithm, parcel to network assignments were also computed using the Infomap algorithm (Figure 2B). This algorithm parcellated the brain into three networks: a visual network (purple), a combined somatomotor/auditory/executive network (blue), and a putative DN (light red). Similar to the Louvain algorithm, the majority of LIM parcels were assigned to the DN. However, in this case several parcels within both OFC and the TP were assigned to the somatomotor/auditory/executive network. These parcels were primarily right-lateralized and included the medial temporal poles, ventral-posterior OFC, and regions within the medial-posterior aspect of the ventral extent of the TP. Results were broadly consistent across runs, with the main difference being a greater assignment of left vATL parcels outside of the DN (Supplementary Figure 2).

### RSFC Map Results

Having provided evidence that the LIM, or subregions thereof, are assigned to the DN in a data-driven manner, we next sought to examine the particular LIM RSFC patterns that underlie these assignments. We first computed RSFC maps for each of the 61 individual LIM parcels in order to comprehensively examine RSFC heterogeneity. Results revealed significant RSFC patterns across parcels, with many exhibiting strong RSFC with the DN and other non-LIM networks, in addition to LIM_A_ and/or LIM_B_. Parcel-wise RSFC maps are shown in Supplementary Figures 7–19. In order to quantitatively summarize these results, we performed data-driven clustering on these maps to assess the presence of LIM subdivisions and examined the differential whole-brain RSFC of distinct clusters. Clustering was first performed on the full LIM (i.e., LIM_A_ and LIM_B_ combined) as defined by the Yeo et al. (2011) 17-network parcellation, and then each of LIM_A_ (TP/vATL) and LIM_B_ (OFC) separately. RSFC maps and contrasts for each of LIM_A_ and LIM_B_ as defined based on Yeo et al. (2011) are shown in Supplementary Figure 3. Results reported here in the main text are on Run 1 data. Results for Run 2 are shown in Supplementary Figures 4–6.

#### Temporal pole and orbitofrontal data-driven clusters.

**Figure 3.**
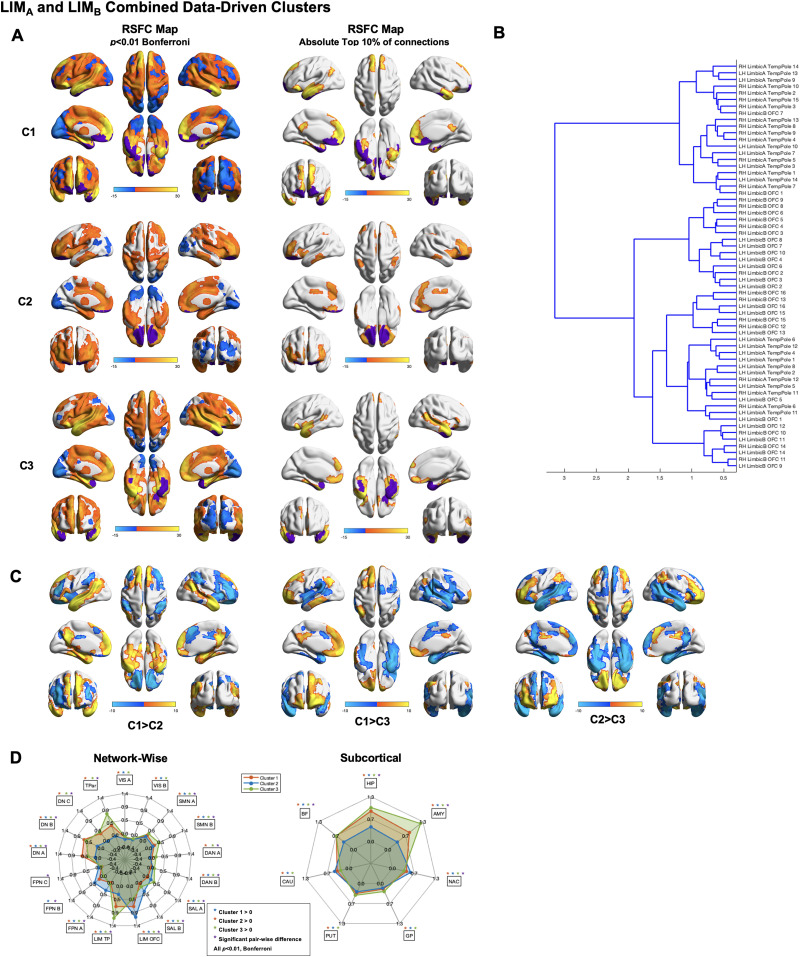
Data-driven clusters revealed by applying Ward clustering to all LIM parcels, spanning both LIM_A_ and LIM_B_. (A) Left: RSFC maps thresholded at *p* < 0.01 Bonferroni for Cluster 1 (C1; top), Cluster 2 (C2; middle), and Cluster 3 (C3; bottom). Right: RSFC maps thresholded at absolute top 10% of connections for Cluster 1 (C1; top), Cluster 2 (C2; middle), and Cluster 3 (C3; bottom). (B) Cluster dendrogram. (C) Between-cluster contrasts. (D) Spider plots displaying network-wise (left) and subcortical (right) between-cluster contrasts. Networks are defined based on the Yeo et al. (2011) 17-network parcellation.

Data-driven clustering performed on the full LIM revealed the presence of multiple clusters and subclusters within this network, supporting our hypothesis of heterogeneity in LIM RSFC (Figure 3B). Findings were highly consistent across runs (Supplementary Figure 4). Qualitative examination of the cluster dendrogram indicates the presence of three primary clusters. Cluster 1 RSFC maps thresholded for the absolute top 10% of connections revealed strong RSFC with regions in bilateral medial prefrontal cortex, anterior, mid, and posterior cingulate, left temporal gyri extending through Wernicke’s area into the supramarginal and angular gyri, right temporal pole, medial temporal lobes, left inferior frontal gyrus, and superior frontal gyrus. Cluster 2 RSFC maps thresholded for the absolute top 10% of connections revealed strong RSFC spanning regions within the bilateral anterior and mid cingulate, dorsomedial prefrontal cortex, inferior temporal gyri, medial temporal lobes, lateral prefrontal cortex, and superior and inferior parietal lobule. Cluster 3 RSFC maps thresholded for the absolute top 10% of connections revealed strong RSFC spanning regions within medial prefrontal cortex, bilateral anterior, mid, and dorsal posterior cingulate, bilateral middle and superior temporal gyri extending into Wernicke’s area, bilateral inferior frontal gyri, and the medial temporal lobes.

Between-subnetwork contrasts revealed a variety of significant differences between clusters. Cluster 1 exhibited significantly greater RSFC than Cluster 2 in the medial prefrontal cortex, posterior cingulate, medial temporal lobes, and primarily left-lateralized superior and middle temporal gyri extending into Wernicke’s area. Cluster 2 exhibited significantly greater RSFC than Cluster 1 in right lateral prefrontal cortex, bilateral inferior frontal gyrus, dorsomedial prefrontal/premotor cortex, and bilateral anterior parietal lobule. Network-wise contrasts between Cluster 1 and Cluster 2 revealed significantly different RSFC in SAL_A_, SAL_B_, LIM_A_, LIM_B_, DN_A_, DN_B_, DN_C_, and TPar. Subcortical contrasts between Cluster 1 and Cluster 2 revealed significantly different RSFC in the HIP, AMY, and BF.

Cluster 1 exhibited significantly greater RSFC than Cluster 3 in the medial prefrontal cortex, posterior cingulate, medial temporal lobes, and primarily left-lateralized superior and middle temporal gyri extending into Wernicke’s area. Cluster 3 exhibited significantly greater RSFC than Cluster 1 in the bilateral superior temporal gyrus and sulcus, bilateral inferior frontal gyrus, inferior somatosensory cortex, Wernicke’s area, and the medial temporal lobes. Network-wise contrasts between Cluster 1 and Cluster 3 revealed significantly different RSFC in SAL_A_, SAL_B_, LIM_A_, LIM_B_, DN_A_, DN_B_, DN_C_, and TPar. Subcortical contrasts between Cluster 1 and Cluster 3 revealed significantly different RSFC in the HIP and AMY.

Cluster 2 exhibited significantly greater RSFC than Cluster 3 in bilateral middle frontal gyrus, parietal lobule, posterior mid cingulate, and ventral OFC. Cluster 3 exhibited significantly greater RSFC than Cluster 2 in anterior/ventral medial prefrontal cortex, posterior cingulate and precuneus, temporal poles, and middle and super temporal gyri extending in Wernicke’s area. Network-wise contrasts between Cluster 2 and Cluster 3 revealed significantly different RSFC in SMN_A_, SMN_B_, DAN_A_, SAL_A_, SAL_B_, LIM_A_, LIM_B_, FPN_A_, FPN_B_, DN_A_, DN_B_, DN_C_, and TPar. Subcortical contrasts between Cluster 2 and Cluster 3 revealed significantly different RSFC in the HIP, AMY, and BF.

#### Temporal pole intra-subnetwork heterogeneity.

**Figure 4.**
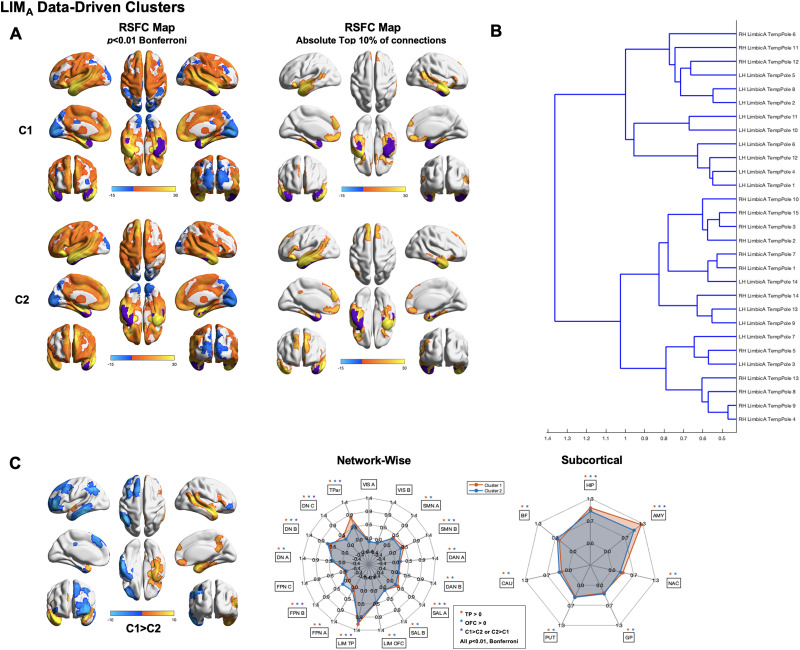
Data-driven clusters revealed by applying Ward clustering to LIM_A_ parcels only. (A) Left: RSFC maps thresholded at *p* < 0.01 Bonferroni for Cluster 1 (C1; top) and Cluster 2 (C2; bottom). Right: RSFC maps thresholded at absolute top 10% of connections for Cluster 1 (C1; top) and Cluster 2 (C2; bottom). (B) Cluster dendrogram. (C) Between-cluster C1 > C2 contrast. (D) Spider plots displaying network-wise (left) and subcortical (right) between-cluster contrasts. Networks are defined based on the Yeo et al. (2011) 17-network parcellation.

Next, we sought to evaluate whether clustering performed separately on each of LIM_A_ and LIM_B_ allows greater sensitivity to finer subdivisions. Data-driven clustering performed on the LIM_A_ revealed the presence of multiple clusters and subclusters (Figure 4B). Findings were highly consistent across runs (Supplementary Figure 5). Qualitative examination of the cluster dendrogram indicates the presence of two primary clusters. Cluster 1 RSFC maps thresholded for the absolute top 10% of connections revealed strong RSFC spanning regions spanning the majority of bilateral temporal lobes. Cluster 2 RSFC maps thresholded for the absolute top 10% of connections revealed strong RSFC spanning the majority of bilateral temporal lobes with a greater extent in the left hemisphere extending into Wernicke’s area and the angular gyrus, medial prefrontal cortex, bilateral inferior, superior, and middle frontal gyri, and portions of anterior, mid, and dorsal-posterior cingulate.

Between-cluster contrasts revealed highly lateralized differences between clusters. Cluster 1 exhibited significantly greater RSFC than Cluster 2 in the right superior temporal gyrus extending from the temporal pole to Wernicke’s area and the angular gyrus, the right inferior temporal gyrus and temporal pole along the ventral surface, the right medial temporal lobe, the right dorsal posterior cingulate extending into the precuneus, and regions within ventromedial and dorsomedial prefrontal cortex. Cluster 2 exhibited significantly greater RSFC than Cluster 1 in left inferior, middle, and superior frontal gyri, left inferior temporal gyrus, left interior parietal lobule/temporoparietal junction, and left dorsomedial prefrontal cortex. Network-wise contrasts between Cluster 1 and Cluster 2 revealed significantly different RSFC in SMN_A_, SAL_A_, LIM_A_, FPN_B_, DN_B_, DN_C_, and TPar. Subcortical contrasts between Cluster 1 and Cluster 2 revealed significantly different RSFC in the HIP and AMY.

#### Orbitofrontal intra-subnetwork heterogeneity.

**Figure 5.**
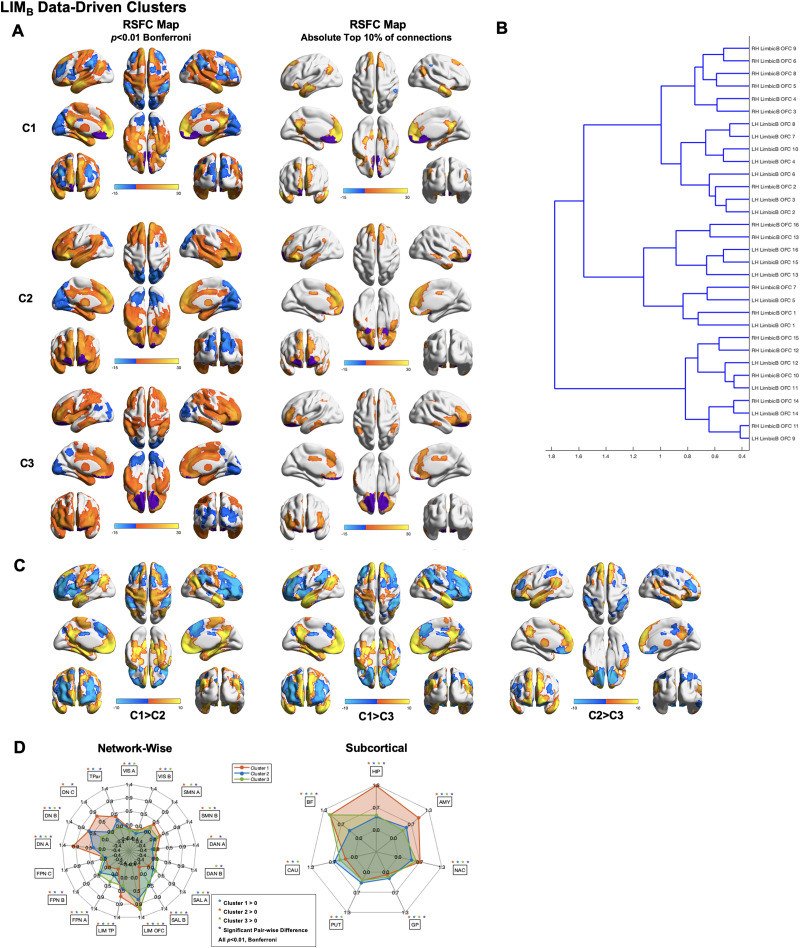
Data-driven clusters revealed by applying Ward clustering to LIM_B_ parcels. (A) Left: RSFC maps thresholded at *p* < 0.01 Bonferroni for Cluster 1 (C1; top), Cluster 2 (C2; middle), and Cluster 3 (C3; bottom). Right: RSFC maps thresholded at absolute top 10% of connections for Cluster 1 (C1; top), Cluster 2 (C2; middle), and Cluster 3 (C3; bottom). (B) Clustering dendrogram. (C) Between-cluster contrasts. (D) Spider plots displaying network-wise (left) and subcortical (right) between-cluster contrasts. Networks are defined based on the Yeo et al. (2011) 17-network parcellation.

Data-driven clustering performed on the LIM_B_ revealed the presence of multiple clusters and subclusters (Figure 5B). Findings were highly consistent across runs (Supplementary Figure 6). Qualitative examination of the cluster dendrogram indicates the presence of three primary clusters. Cluster 1 RSFC maps thresholded for the absolute top 10% of connections revealed strong RSFC spanning regions within the bilateral medial prefrontal cortex, anterior and posterior cingulate, temporal poles, posterior inferior parietal lobule, medial temporal lobes, inferior and superior frontal gyri, and superior somatomotor cortex. Cluster 2 RSFC maps thresholded for the absolute top 10% of connections revealed strong RSFC spanning regions within the bilateral medial prefrontal cortex, bilateral anterior and mid cingulate, left dorsal posterior cingulate, temporal poles, left inferior parietal lobule, medial temporal lobes, and bilateral superior, middle, and inferior frontal gyri. Cluster 3 RSFC maps thresholded for the absolute top 10% of connections revealed strong RSFC spanning the cingulate gyrus extending from its subgenual portion to immediately anterior to its dorsal-posterior portion, bilateral inferior and middle frontal gyri, bilateral parietal lobules, dorsomedial prefrontal cortex, and medial temporal lobes.

Between-subnetwork contrasts revealed a variety of significant differences between clusters. Cluster 1 exhibited significantly greater RSFC than Cluster 2 in the medial prefrontal cortex, posterior cingulate, medial temporal lobes, bilateral somatomotor cortex, and bilateral temporal poles extending along the ventral surface of the temporal lobes/inferior frontal gyri. Cluster 2 exhibited significantly greater RSFC than Cluster 1 in bilateral superior, middle, and inferior frontal gyri, dorsomedial prefrontal/premotor cortex, and anterior superior parietal lobule. Network-wise contrasts between Cluster 1 and Cluster 2 revealed significantly different RSFC in SMN_A_, SMN_B_, DAN_A_, SAL_A_, SAL_B_, LIM_A_, LIM_B_, FPN_A_, FPN_B_, DN_A_, and DN_C_. Subcortical contrasts between Cluster 1 and Cluster 2 revealed significantly different RSFC in the HIP, AMY, NAC, and BF.

Cluster 1 exhibited significantly greater RSFC than Cluster 3 in the medial prefrontal cortex, posterior cingulate, medial temporal lobes, and bilateral temporal poles extending along the ventral surface of the temporal lobes/inferior frontal gyri. Cluster 3 exhibited significantly greater RSFC than Cluster 1 in bilateral superior, middle, and inferior frontal gyri, dorsomedial prefrontal/premotor cortex, and anterior superior parietal lobule. Network-wise contrasts between Cluster 1 and Cluster 3 revealed significantly different RSFC in SMN_A_, SMN_B_, DAN_A_, SAL_A_, SAL_B_, LIM_A_, LIM_B_, FPN_A_, FPN_B_, DN_A_, DN_B_, and DN_C_. Subcortical contrasts between Cluster 1 and Cluster 3 revealed significantly different RSFC in the HIP and AMY.

Cluster 2 exhibited significantly greater RSFC than Cluster 3 in bilateral anterior and dorsal medial prefrontal cortex, dorsal posterior cingulate cortex, bilateral temporal poles, bilateral inferior parietal lobule/angular gyri, bilateral inferior frontal gyri, and the left superior frontal gyrus. Cluster 3 exhibited significantly greater RSFC than Cluster 2 in ventromedial and orbital prefrontal cortex, bilateral middle frontal gyri, bilateral superior parietal lobule, right precuneus, and right posterior-mid cingulate. Network-wise contrasts between Cluster 2 and Cluster 3 revealed significantly different RSFC in SMN_A_, SMN_B_, DAN_A_, LIM_B_, FPN_A_, DN_A_, DN_B_, and TPar. Subcortical contrasts between Cluster 2 and Cluster 3 revealed significantly different RSFC in the BF and CAU.

## DISCUSSION

In the present study, we leveraged a multi-echo resting-state fMRI dataset with strong whole-brain signal coverage, including orbitofrontal and anterior temporal regions, to characterize the large-scale network organization and whole-brain RSFC of the LIM as defined by the Yeo et al. (2011) 17-network parcellation. This includes the two putative LIM subnetworks: LIM_A_ (comprising the TP and spatially adjacent vATL), and LIM_B_ (comprising the OFC). Our primary goal was to evaluate whether the LIM, or subregions thereof, may be plausibly construed to be part of the DN, and, in addition, to examine intra-LIM heterogeneity in whole-brain RSFC via data-driven clustering and RSFC mapping. Data-driven modularity results with the Louvain algorithm supported the inclusion of the LIM within the DN: With the exception of parcels within the right medial temporal pole, all LIM parcels were assigned to the DN across the resolutions examined. These results were supported by an additional community detection approach, the Infomap algorithm, which also revealed that most LIM parcels were assigned to the DN. Data-driven clustering results indicated the presence of RSFC heterogeneity across the LIM as a whole, as well as within each of LIM_A_ and LIM_B_ separately. Distinct parcel clusters within the LIM exhibited differential RSFC with cortical and subcortical areas across the brain, with medial OFC and left-lateralized TP regions exhibiting the greatest RSFC with the DN, in a subnetwork-specific manner. Taken together, both the data-driven clustering and a priori intra-LIM functional connectivity findings provide strong evidence that a large proportion of the LIM network—particularly in the left hemisphere—may in fact be an extension of the DN.

Modularity results from both algorithms assigned the majority of LIM parcels to the same module/network as putative DN regions. This indicates that most LIM regions exhibit strong RSFC with DN regions and that their RSFC with each other is not sufficiently greater to afford their segregation into a distinct network. LIM parcels assigned outside of the DN were assigned to modules corresponding to executive (frontoparietal/dorsal attention/salience) and/or somatomotor networks. These parcels were exclusively in the right medial TP for the Louvain algorithm and, for the Infomap algorithm, comprised bilateral medial TPs, a mostly right-lateralized set of parcels in the posterior OFC and an expanse of the right vATL spanning from its anterior-medial to posterior-lateral extent. This set of non-DN parcels within the vATL revealed by the Infomap algorithm was found to be more bilaterally localized for Run 2 (Supplementary Figure 1). The functional differentiation of the posteromedial versus anterolateral vATL has been highlighted in past task-based fMRI research (Binney et al., 2016; Humphreys et al., 2015; Jackson et al., 2016). This work has argued that the vATL can be characterized in terms of a gradient from basic and modality-specific semantic representations within its posteromedial aspect, to complex and modality-general representations within its anterolateral aspect (Binney et al., 2016; Humphreys et al., 2015; Jackson et al., 2016). According to this work, the anterolateral vATL—which is the region of the vATL consistently assigned to the DN in the present study—represents the apex of the ATL’s processing hierarchy, acting as a multi-modal integrative hub (Binney et al., 2016). However, a previous ATL functional mapping investigation, which utilized an fMRI acquisition that was optimized for TSNR in the ATL, challenged this view (Persichetti et al., 2021). This study parcellated the ATL into 34 distinct parcels, and provided evidence of clusters that exhibit differential RSFC with other large-scale networks—similar to the present findings. Notably, their findings indicated discrete and abrupt transitions in RSFC between spatially contiguous regions of the ATL as opposed to a gradient, and also did not reveal parcels with the diverse RSFC profile expected of a “domain-general hub” (Persichetti et al., 2021). This study additionally found several ATL subregions that exhibit strong FC with the DN—particularly the DN_B_. This set of findings is highly consistent with the present results, which also indicate abrupt transitions in RSFC across ATL regions and strong RSFC with the DN_B_ in particular. The present results therefore align with this previous study in pushing back against gradient/multi-modal hub accounts of the ATL and, instead, highlight that much of the vATL may predominantly act in concert with the DN_B_ in support of social and semantic processes. Our results also expand on this past work by providing more clear evidence that left-lateralized and anterolateral vATL in particular may be most associated with the DN.

The parcel-wise RSFC maps (shown in the Supporting Information) and their summarization in the form of data-driven clusters provided deeper insight into the differential RSFC patterns of LIM subdivisions and how these might underpin the modularity assignments. Notably, the parcels assigned outside of the DN by the Infomap algorithm significantly overlap with Cluster 3 from the combined LIM_A_-LIM_B_ clustering analysis and Cluster 1 from the LIM_A_ clustering analysis. These two clusters both comprise the majority of the right vATL, as well as portions of the bilateral medial TPs. In the case of the LIM_A_-LIM_B_ clustering analysis, this cluster was differentiated from a cluster that combined the left TP and vATL with medial OFC regions and that exhibited significantly greater RSFC with the DN. In the case of the LIM_A_ clustering analysis, this cluster was differentiated from a cluster that comprised the left medial TP and vATL and that exhibited greater RSFC with a left-lateralized set of regions spanning the DN and the semantic control system (Binder et al., 2009; Jackson, 2021). These clustering results therefore suggest that left-lateralized TP/vATL regions may be more associated with the DN, potentially via their involvement in semantic processing.

The present results are also informed by recent studies applying a “precision mapping” approach to characterize brain network—and, specifically, DN—functional organization (Braga & Buckner, 2017). Leveraging dense sampling at the within-subject level, this work has revealed spatially fine-grained features of network organization that are obscured at the group level owing to interindividual differences in functional neuroanatomy. With respect to the DN, it has provided evidence that the DN might be composed of two distinct networks: one that includes parahippocampal and retrosplenial cortex and is preferentially involved in episodic memory (“Network A”), and one that includes the temporoparietal junction and is preferentially involved in theory of mind/social cognition (“Network B”; Braga & Buckner, 2017; DiNicola et al., 2020). Consistent with present results, Network B includes regions of the ventromedial prefrontal/medial orbitofrontal cortex that are a part of LIM_B_. Additional work by this group also revealed a left-lateralized language network that is distinct from the DN, and which overlaps with a region of the TP that is adjacent/superior to the anterolateral LIM parcels found to be associated with the DN here (Braga et al., 2020). Individualized approaches to estimating functional brain regions to accommodate interindividual differences in brain network architecture (Uddin et al., 2023), such as group prior individual parcellation (GPIP; Chong et al., 2017), may provide a more refined illumination of network associations for ATL and OFC. In order to reduce the complexity of variation in seed and cortical targets for RSFC, we leveraged a standardized approach to delineating RSFC between LIM parcels and cortex. Yet further work is needed to ascertain whether the attribution of some of these parcels to the DN in the present work are due to the limitations of group-level averaging.

Interestingly, relative to left-lateralized clusters dominated by LIM_A_, the strongly right-lateralized LIM_A_-LIM_B_ cluster comprising the TP, vATL, and posterior OFC exhibited greater RSFC with the bilateral superior temporal gyri and sulci, as well as the inferior frontal gyrus and insula. This pattern has been found in social and nonverbal semantic processing task-fMRI investigations that have argued for a right hemisphere bias in the ATL for the processing of social concepts and nonverbal stimuli (Kumfor et al., 2016; Skipper et al., 2011; Zahn et al., 2007). As such, the hemispheric dissociation in RSFC observed here—also partially mirrored in the modularity results—may reflect the distinction between a left-lateralized TP/vATL network that is closely related to the semantic system, which is predominantly involved in verbal and nonsocial semantic processing, and a right-lateralized TP/vATL network more involved in nonverbal and social semantic processing. It should be noted here, however, that previous studies of the RSFC of TP and vATL subdivisions have not found strong evidence for lateralization effects (Fan et al., 2014; Jackson et al., 2016), and multiple fMRI studies have found strong bilateral activation for social concepts and stimuli (Binney et al., 2016; Jackson et al., 2018). That said, it is possible that the former may be due to the relatively poor signal quality of LIM regions in past fMRI investigations. With respect to previously observed bilateral activation, strong evidence from lesion studies and patients with lateralized semantic dementia suggests that some degree of right-lateralized specificity for social concepts may indeed exist (Pobric et al., 2016; Rice et al., 2018; Snowden et al., 2018), and it is ultimately likely that each hemisphere has a partially specialized and graded contribution across semantic tasks (Ralph et al., 2017; Rice et al., 2018). The high TSNR in the present study may have provided greater sensitivity to hemispheric differences in TP/vATL RSFC.

DN-associated clusters of LIM_A_ and LIM_B_ did not exhibit uniform RSFC across the entire DN, but, rather, showed DN-subnetwork-specific patterns. Both LIM_A_ clusters were most associated with DN_B_ in particular, with strong RSFC also present for DN_A_. The association between the TP/vATL and the DN_B_ is consistent with this subnetwork’s spatial overlap with the semantic processing system, and its role in social cognitive processes for which the temporal lobes are consistently co-recruited (Binder et al., 2009; Olson et al., 2013; Spreng & Andrews-Hanna, 2015). Indeed, the investigation that introduced the tripartite subnetwork organization of the DN included the TP as a component of DN_B_ (Andrews-Hanna et al., 2010). LIM_A_ clusters were also particularly strongly connected to the TPar network (Yeo et al., 2011), which encompasses the superior temporal gyri and sulci, including primary auditory cortex and extending posteriorly into Wernicke’s area and anterior visual association regions. TPar RSFC was particularly strong for the predominantly right-lateralized LIM_A_ Cluster 1. This can be seen to be consistent with the findings discussed above, supporting a role for right superior temporal regions in right-lateralized social cognitive and nonverbal (i.e., pictorial) semantic processes (Kumfor et al., 2016; Pobric et al., 2016; Rice et al., 2018; Skipper et al., 2011; Snowden et al., 2018; Zahn et al., 2007). In contrast, LIM_B_ Cluster 1 (medial-most OFC) was most associated with DN_A_ and DN_C_. This directly aligns with a recent large-scale RSFC study of the OFC that found that medial subregions exhibited RSFC patterns most overlapping with the DN midline (i.e., DN_A_) and the medial temporal lobes (DN_C_; Du et al., 2020). Moreover, the functional differentiation of medial versus lateral OFC is supported by a large multi-species literature of task-based studies (Kringelbach & Rolls, 2003; Rolls et al., 2020; Schultz et al., 2000; Xie et al., 2021), as well as additional functional and anatomical parcellation studies (Hsu et al., 2020; Rolls et al., 2020; Rolls et al., 2023). It remains unclear, however, why medial OFC regions in particular, which mediate positive reward value, should be more connected to the DN than lateral OFC regions, which are involved in the negative reward value/the denial of an expected reward (Rolls et al., 2020; Small et al., 2001). LIM_B_ Cluster 2, which comprised a small number of parcels within the most anterior and posterior aspects of the OFC, was most associated with DN_B_. This also aligns with the results of the above-mentioned large scale RSFC study, in which similarly located parcels exhibited broadly similar RSFC patterns (Du et al., 2020). The results here extend these findings by highlighting how these spatially noncontiguous regions form a functionally dissociable subnetwork within the OFC, with a specific pattern of strong DN_B_ RSFC. Future work is needed to investigate the cognitive/behavioral functionality of this distributed cluster. Finally, Cluster 3 (the remainder of the OFC) was not significantly associated with the DN—exhibiting minimal RSFC across DN subsystems. Instead, Cluster 3 was most associated with FPN and SAL subnetworks, potentially in line with the OFC’s documented role in reward-based cognitive control (Dixon & Christoff, 2012; Dixon et al., 2017). Overall, results based on RSFC clustering indicate that several subdivisions of LIM exhibit significant RSFC with the DN in a subsystem-specific manner. These results underscore the notion that the LIM is likely not a unified and distinct network, but is a collection of regions that show differential patterns of whole-brain RSFC—most prominently with particular DN subnetworks—to an extent that suggests membership in these subnetworks.

All LIM subdivisions exhibited significant RSFC with the subcortical regions examined—which included the hippocampus, amygdala, nucleus accumbens, globus pallidus, putamen, caudate, and basal forebrain—indicating strong interconnectivity across limbic, cortical, and subcortical regions. Of note, the clusters that exhibited the greatest RSFC with the DN included the hippocampus, amygdala, basal forebrain, and to a lesser extent the nucleus accumbens. This is consistent with past work arguing for the inclusion of these regions as part of the DN and, in the case of the basal forebrain, for an important neuromodulatory role of ascending subcortical inputs in DN function (Alves et al., 2019; Buckner & DiNicola, 2019; Bzdok et al., 2013; Li et al., 2021; Markello et al., 2018). This highlights the limitations of previous cortico-centric—and non-limbic—conceptions of the DN and suggests the presence of a combined cortical-limbic-subcortical extended DN.

In conclusion, by leveraging multi-echo fMRI data that provide strong TSNR in LIM regions, we found support for the hypothesis that the LIM is an extension of the DN. Our results also highlighted the distributed and heterogenous nature of LIM RSFC, indicating the presence of LIM subregions with distinct RSFC across DN subnetworks and other large-scale networks. Among LIM regions, we found that the medial OFC and left TP/vATL were most associated with the DN and, more specifically, that medial OFC was primarily associated with DN_A_ and DN_C_, while the left TP/vATL was primarily associated with DN_B_. Our findings are consistent with and extend past task-based and RSFC investigations of the TP, vATL, and OFC, providing novel evidence on the large-scale network affiliations of these regions. This study contributes to the growing literature expanding the set of regions conceived of as constituents of the DN and suggests that past accounts of a distinct LIM may have been based on poor signal quality and relatively unreliable functional mapping.

## SUPPORTING INFORMATION

Supporting information for this article is available at https://doi.org/10.1162/netn_a_00385.

## AUTHOR CONTRIBUTIONS

Manesh Girn: Conceptualization; Formal analysis; Investigation; Methodology; Visualization; Writing – original draft; Writing – review & editing. Roni Setton: Data curation; Methodology; Validation; Writing – review & editing. Gary Turner: Funding acquisition; Writing – review & editing. R. Nathan Spreng: Conceptualization; Funding acquisition; Supervision; Writing – original draft; Writing – review & editing.

## FUNDING INFORMATION

This work was supported in part by National Institutes of Health Grant 1S10RR025145 and by Natural Sciences and Engineering Research Council of Canada and Canadian Institutes of Health Research grants to RNS, a research scholar supported by the Fonds de la Recherche du Quebec – Santé (FRQS).

## Supplementary Material



## TECHNICAL TERMS

Resting-state functional connectivity (RSFC): An fMRI-based approach that characterizes large-scale functional brain networks based on correlations between brain regions in their spontaneous low-frequency activity.
Large-scale functional network: A set of brain regions that preferentially exhibit functional connectivity with each other and/or coactivate to perform shared functions.
Default network: A large-scale functional brain network including posterior cingulate and medial prefrontal cortex, medial and lateral temporal lobes, and angular gyrus. By some characterizations, the default network comprises multiple subsystems.
Temporal signal-to-noise ratio (TSNR): A metric that assesses data reliability at each spatial location (voxel, vertex), computed as the mean signal intensity over time divided by the standard deviation of the time course.
Multi-echo fMRI (ME-fMRI): T2*-weighted imaging sensitive to BOLD signal that collects data at multiple echo times, resulting in multiple volumes with varying levels of contrast acquired per radio frequency pulse.
Echo time (TE): A pulse sequence parameter providing the timing within a repetition time wherein a whole-brain volume is acquired relative to a single radio frequency pulse. In the current study, the TEs are 13.7 ms, 30 ms, and 47 ms.
Cluster analysis: A data-driven statistical technique that organizes multiple items into groups based on their statistical properties, usually on the basis of quantitative similarity. In the current application, multiple patterns of seed-based RSFC are grouped into subnetworks.
Topography: The spatial distribution of features within an organ; here, the spatial distribution of brain regions that comprise large-scale brain networks.
Subsystem: A subset of regions within a large-scale network that can be differentiated based on their functional connectivity profile and functional affiliations.
Extended default network: A broad characterization of the default network that comprises submultiple subsystems that have dissociable neuroanatomic functions, including memory, social cognition, language, and emotional processing.
