# Supplementary material for: The “limbic network,” comprising orbitofrontal and anterior temporal cortex, is part of an extended default network: Evidence from multi-echo fMRI
Source: Netw Neurosci. 2024 Oct 1;8(3):860–82. doi: 10.1162/netn_a_00385 (PMC11398723; doi:10.1162/netn_a_00385)
Supplement: Supplementary file 1 [file netn-8-3-860-s001.pdf]

Supplementary Material

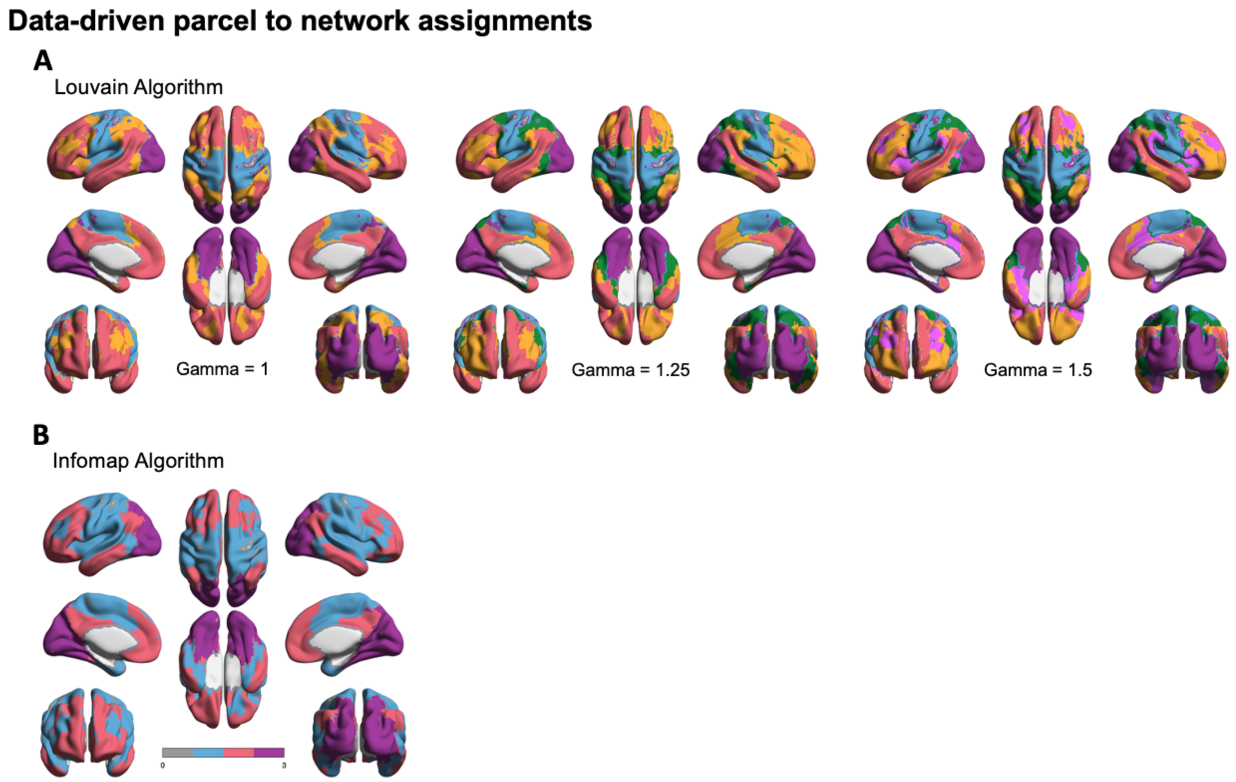

Supplementary Figure 1. Modularity results for Run 2. **(A)** Data-driven network assignments based on the Louvain modularity algorithm, at three values of the gamma resolution parameter. **(B)** Data-driven network assignments based on the Infomap modularity algorithm. Networks are colored according to their putative corresponding large-scale network according to the Yeo et al., (2011) 7-network assignments.

Louvain parcel to network assignments at higher resolution

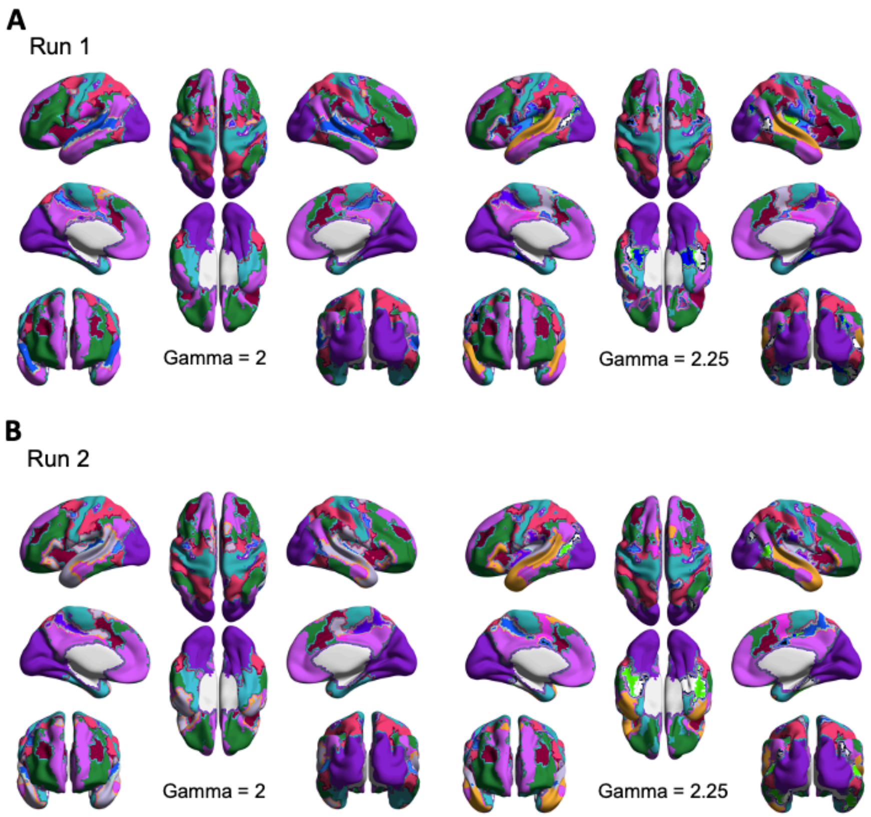

Supplementary Figure 2. Louvain modularity results at higher levels of the gamma resolution parameter **(A)** Louvain network assignments for Run 1 at gamma=2 (left; X networks) and at gamma=2.25 (right; X networks). **(B)** Louvain network assignments for Run 2 at gamma=2 (left; X networks) and at gamma=2.25 (right; X networks).

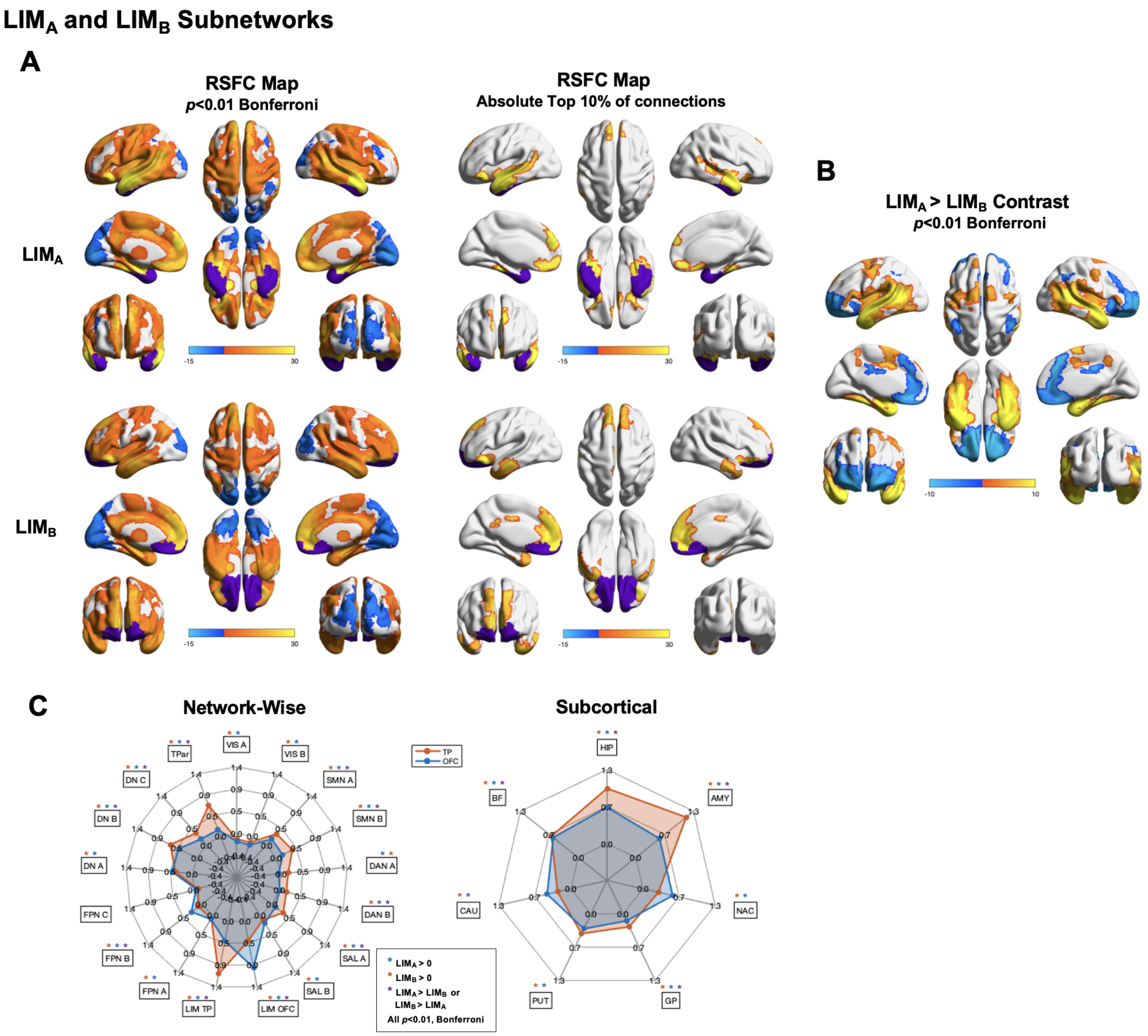

Supplementary Figure 3. RSFC maps for LIM$_A$ and LIM$_B$, as defined by the Yeo et al. (2011) 17-network assignments, averaged across runs. **(A) Left:** RSFC maps thresholded at p<0.01 Bonferroni for Cluster 1 (C1; Top) and Cluster 2 (C2; Bottom). **Right:** RSFC maps thresholded at top absolute 10% of connections for Cluster 1 (C1; Top) and Cluster 2 (C2; Bottom **(B)** Cluster dendrogram. **(C)** Between-cluster C1>C2 contrast. **(D)** Spider plots displaying network-wise (Left) and subcortical (Right) between-cluster contrasts. Networks are defined based on the Yeo et al. (2011) 17-network assignments.

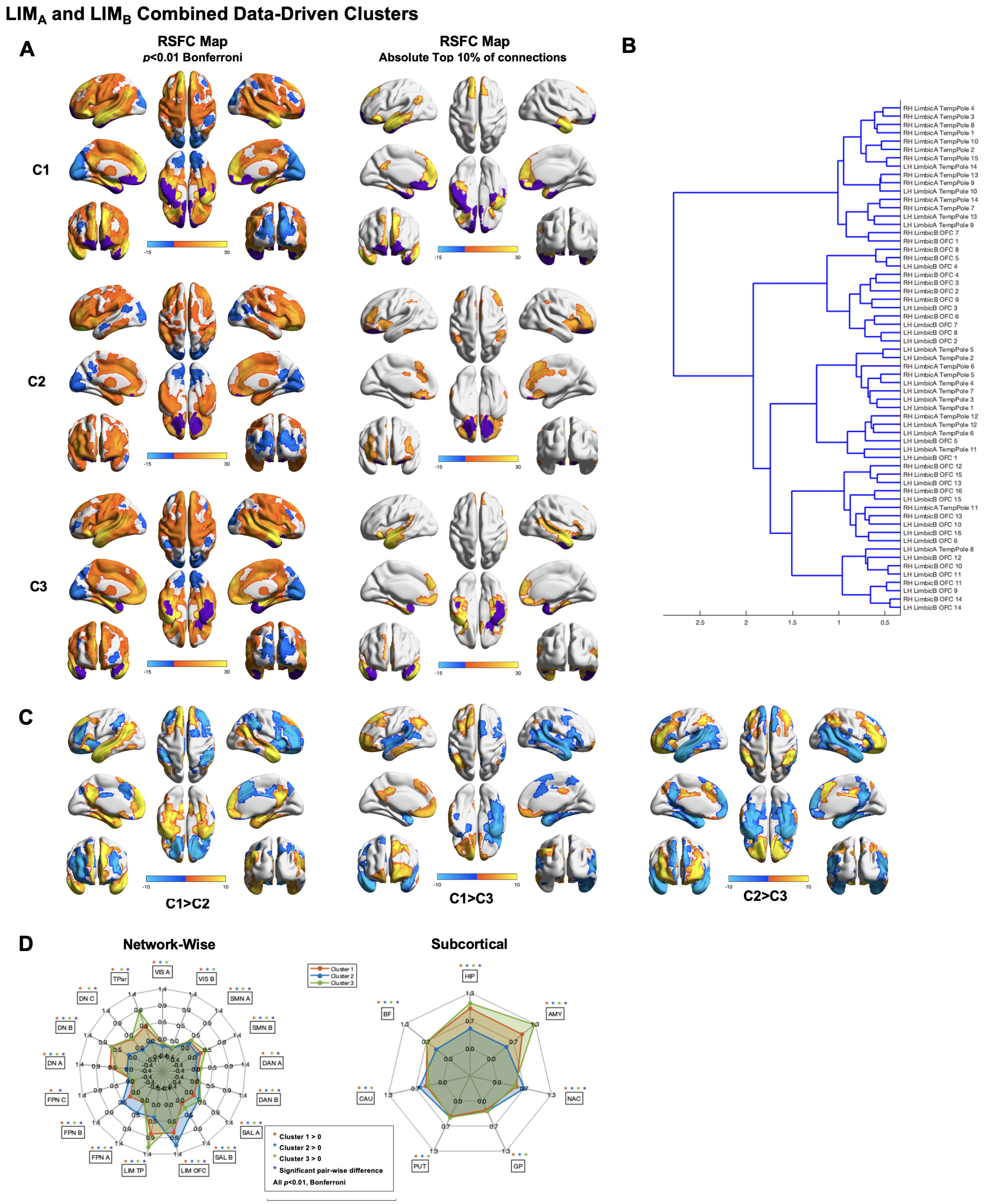

Supplementary Figure 4. Run 2 data-driven clusters revealed by applying Ward clustering to all LIM parcels, spanning both LIM_A and LIM_B **(A) Left:** RSFC maps thresholded at p<0.01 Bonferroni for Cluster 1 (C1; Top), Cluster 2 (C2; Middle), and Cluster 3 (C3; Bottom). **Right:** RSFC maps thresholded at top absolute 10% of connections for Cluster 1 (C1; Top), Cluster 2 (C2; Middle), and Cluster 3 (C3; Bottom). **(B)** Cluster dendrogram. **(C)**

Between-cluster contrasts. **(D)** Spider plots displaying network-wise (Left) and subcortical (Right) between-cluster contrasts. Networks are defined based on the Yeo et al. (2011) 17-network assignments.

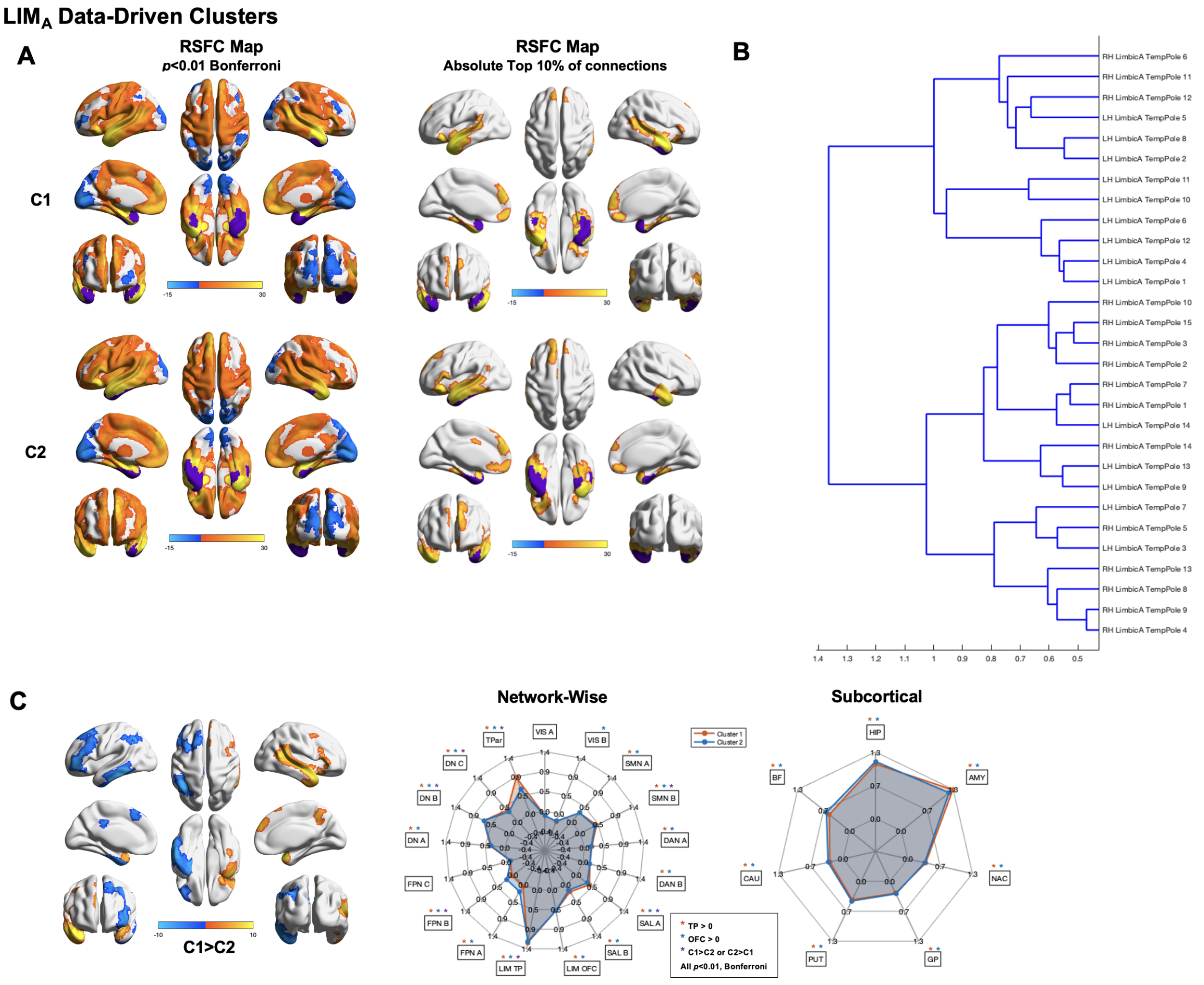

Supplementary Figure 5. Run 2 data-driven clusters revealed by applying Ward clustering to LIM_A parcels only **(A) Left:** RSFC maps thresholded at p<0.01 Bonferroni for Cluster 1 (C1; Top) and Cluster 2 (C2; Bottom). **Right:** RSFC maps thresholded at top absolute 10% of connections for Cluster 1 (C1; Top) and Cluster 2 (C2; Bottom **(B)** Cluster dendrogram. **(C)** Between-cluster C1>C2 contrast. **(D)** Spider plots displaying network-wise (Left) and subcortical (Right) between-cluster contrasts. Networks are defined based on the Yeo et al. (2011) 17-network assignments.

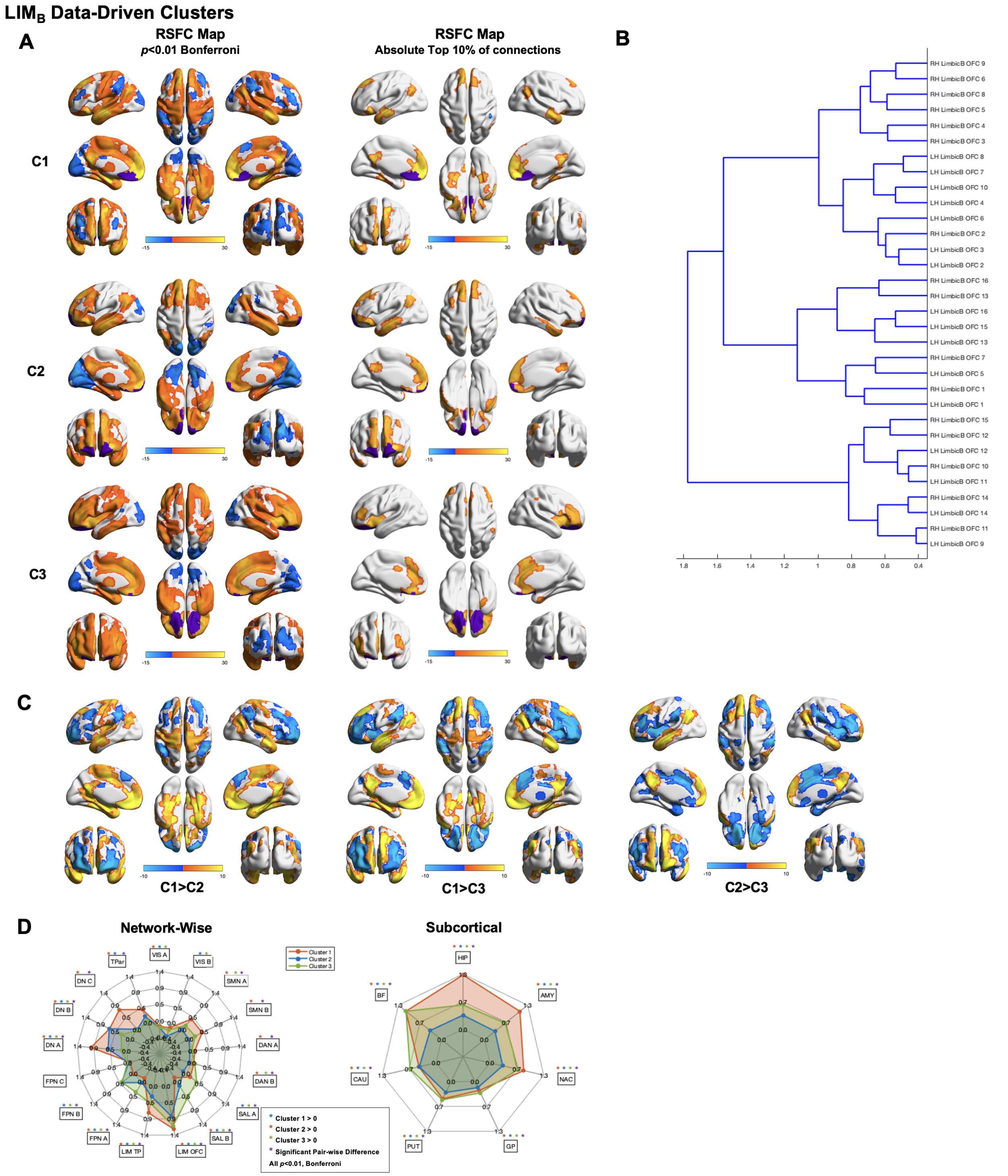

Supplementary Figure 6. Run 2 data-driven clusters revealed by applying Ward clustering to LIMB parcels **(A) Left:** RSFC maps thresholded at p<0.01 Bonferroni for Cluster 1 (C1; Top), Cluster 2 (C2; Middle), and Cluster 3 (C3; Bottom). **Right:** RSFC maps thresholded at top absolute 10% of connections for Cluster 1 (C1; Top), Cluster 2 (C2; Middle), and Cluster 3 (C3; Bottom). **(B)** Clustering dendrogram. **(C)** Between-cluster contrasts. **(D)** Spider plots displaying network-wise (Left) and subcortical (Right) between-cluster contrasts. Networks are defined based on the Yeo et al. (2011) 17-network assignments.

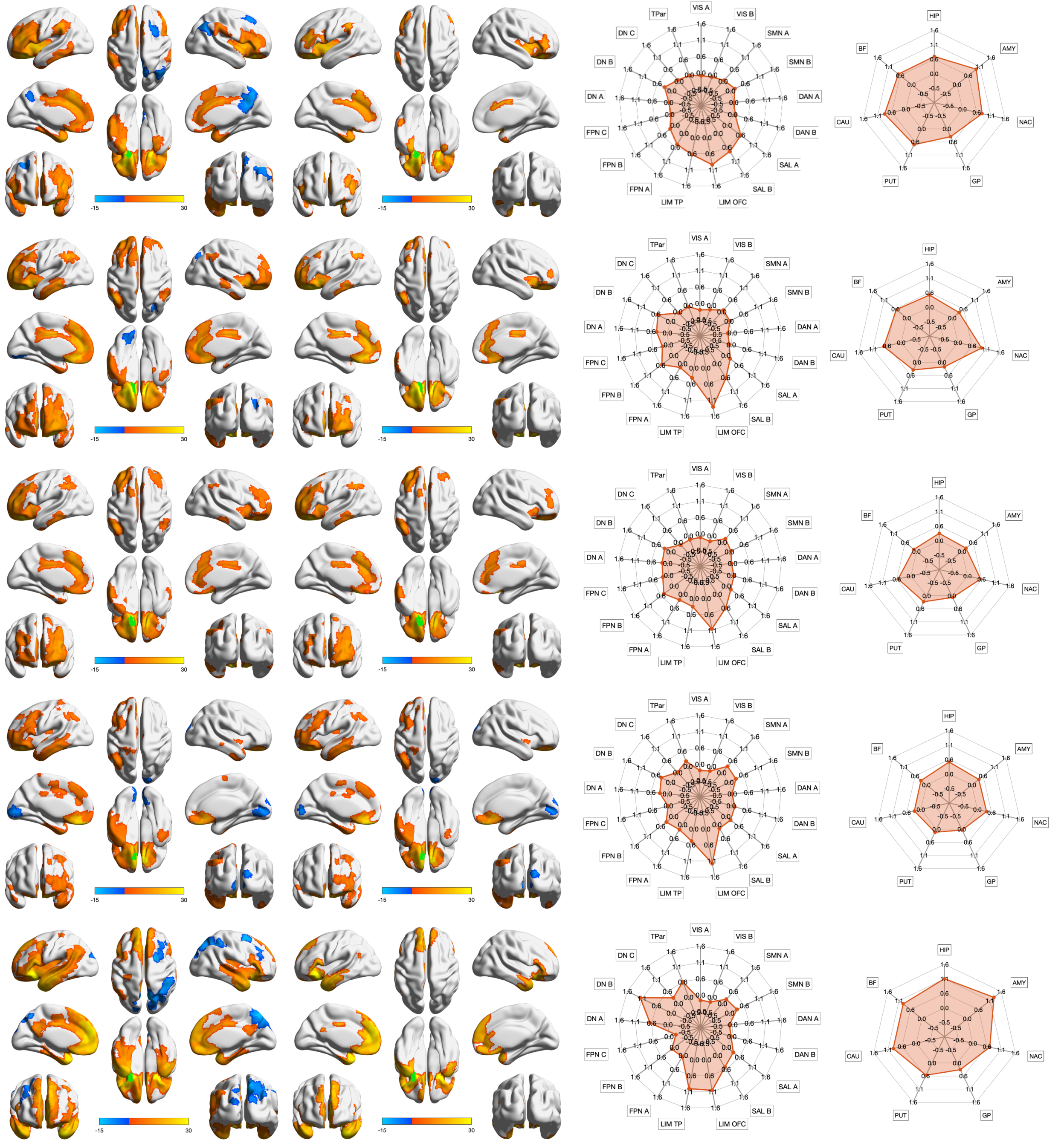

Supplementary Figure 7. Correlation maps for LIM parcels 1-5 (LIM_B), as defined based on the Schaefer-Yeo 1000 region cortical parcellation, averaged across runs. Parcels are shown in light green. Each row corresponds to a given parcel. (Left-Left) The correlation map thresholded at p<0.01, Bonferroni. (Left-Middle) The correlation map thresholded at 90% sparsity. (Right-Middle) Spider plot indicating mean correlation with each of the Yeo et al. (2011) 17-network assignments. (Right-Right) Spider plot indicating mean correlation with each examined subcortical region as per Tian et al. (2020).

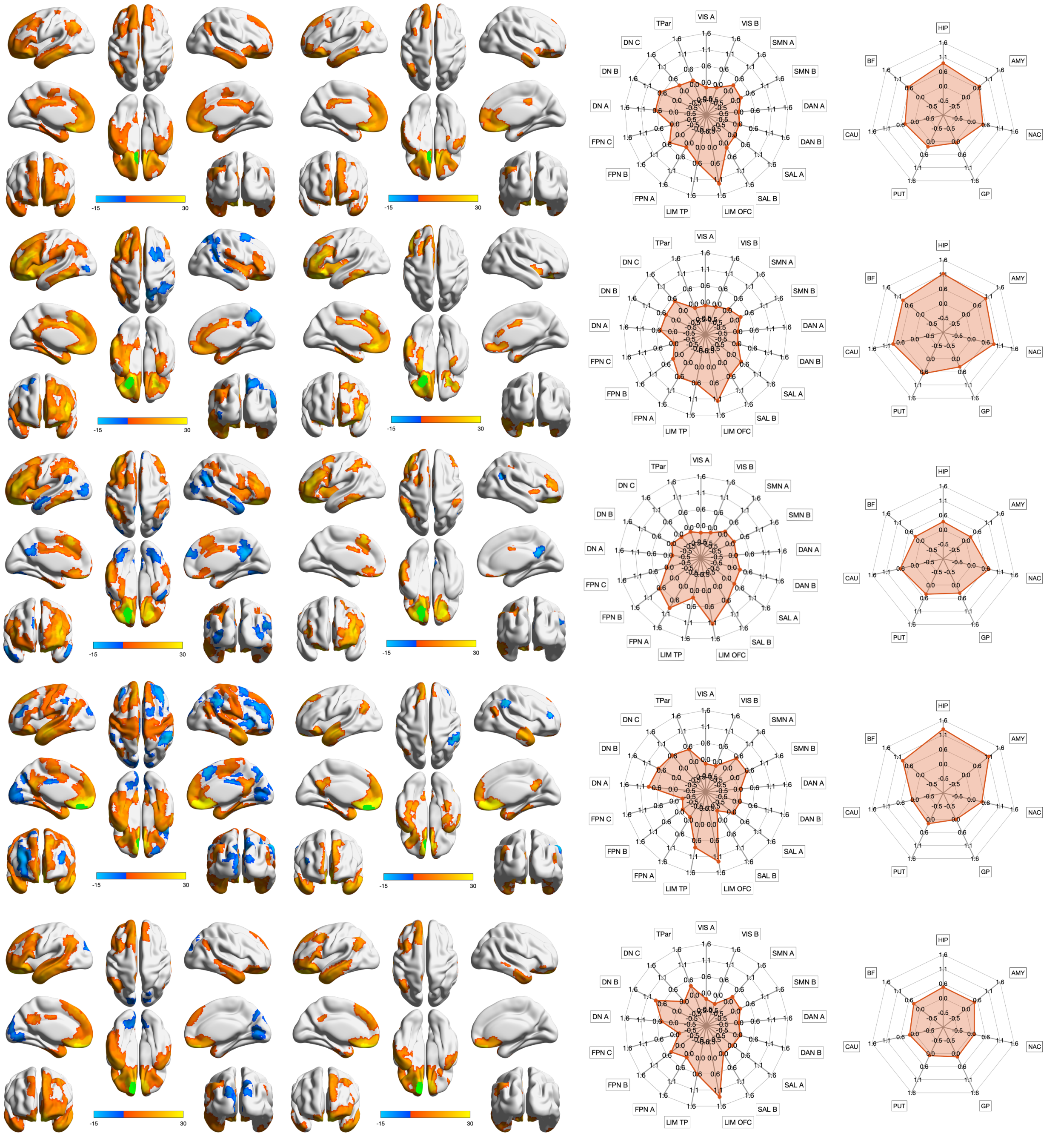

Supplementary Figure 8. Correlation maps for LIM parcels 6-10 (LIM_B), as defined based on the Schaefer-Yeo 1000 region cortical parcellation, averaged across runs. Parcels are shown in light green. Each row corresponds to a given parcel. (Left-Left) The correlation map thresholded at p<0.01, Bonferroni. (Left-Middle) The correlation map thresholded at 90% sparsity. (Right-Middle) Spider plot indicating mean correlation with each of the Yeo et al. (2011) 17-network assignments. (Right-Right) Spider plot indicating mean correlation with each examined subcortical region as per Tian et al. (2020).

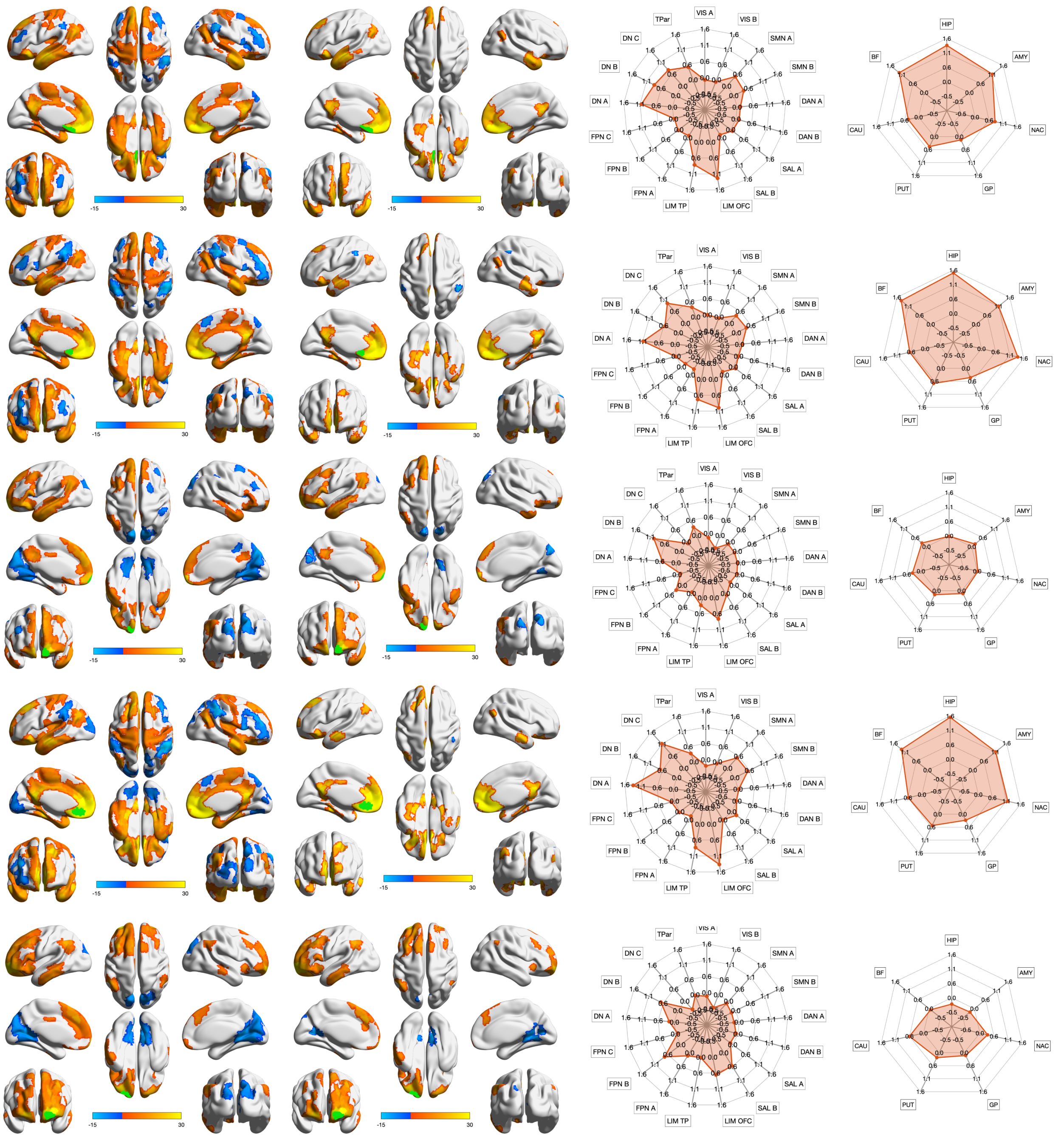

Supplementary Figure 9. Correlation maps for LIM parcels 11-15 (LIM$_{B}$), as defined based on the Schaefer-Yeo 1000 region cortical parcellation, averaged across runs. Parcels are shown in light green. Each row corresponds to a given parcel. (Left-Left) The correlation map thresholded at p<0.01, Bonferroni. (Left-Middle) The correlation map thresholded at 90% sparsity. (Right-Middle) Spider plot indicating mean correlation with each of the Yeo et al. (2011) 17-network assignments. (Right-Right) Spider plot indicating mean correlation with each examined subcortical region as per Tian et al. (2020).

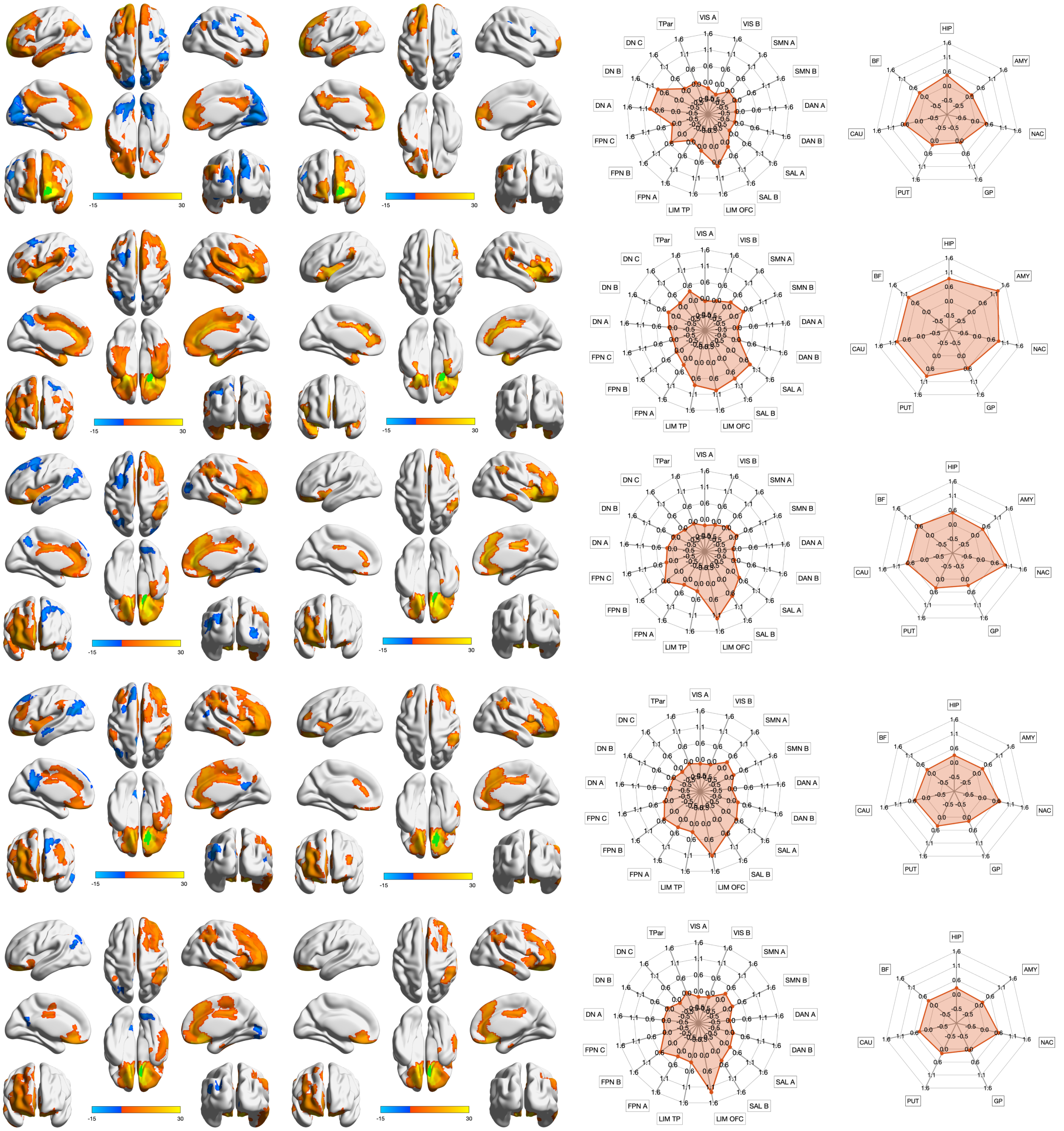

Supplementary Figure 10. Correlation maps for LIM parcels 16-20 (LIM$_B$), as defined based on the Schaefer-Yeo 1000 region cortical parcellation, averaged across runs. Parcels are shown in light green. Each row corresponds to a given parcel. (Left-Left) The correlation map thresholded at $p<0.01$, Bonferroni. (Left-Middle) The correlation map thresholded at 90% sparsity. (Right-Middle) Spider plot indicating mean correlation with each of the Yeo et al. (2011) 17-network assignments. (Right-Right) Spider plot indicating mean correlation with each examined subcortical region as per Tian et al. (2020).

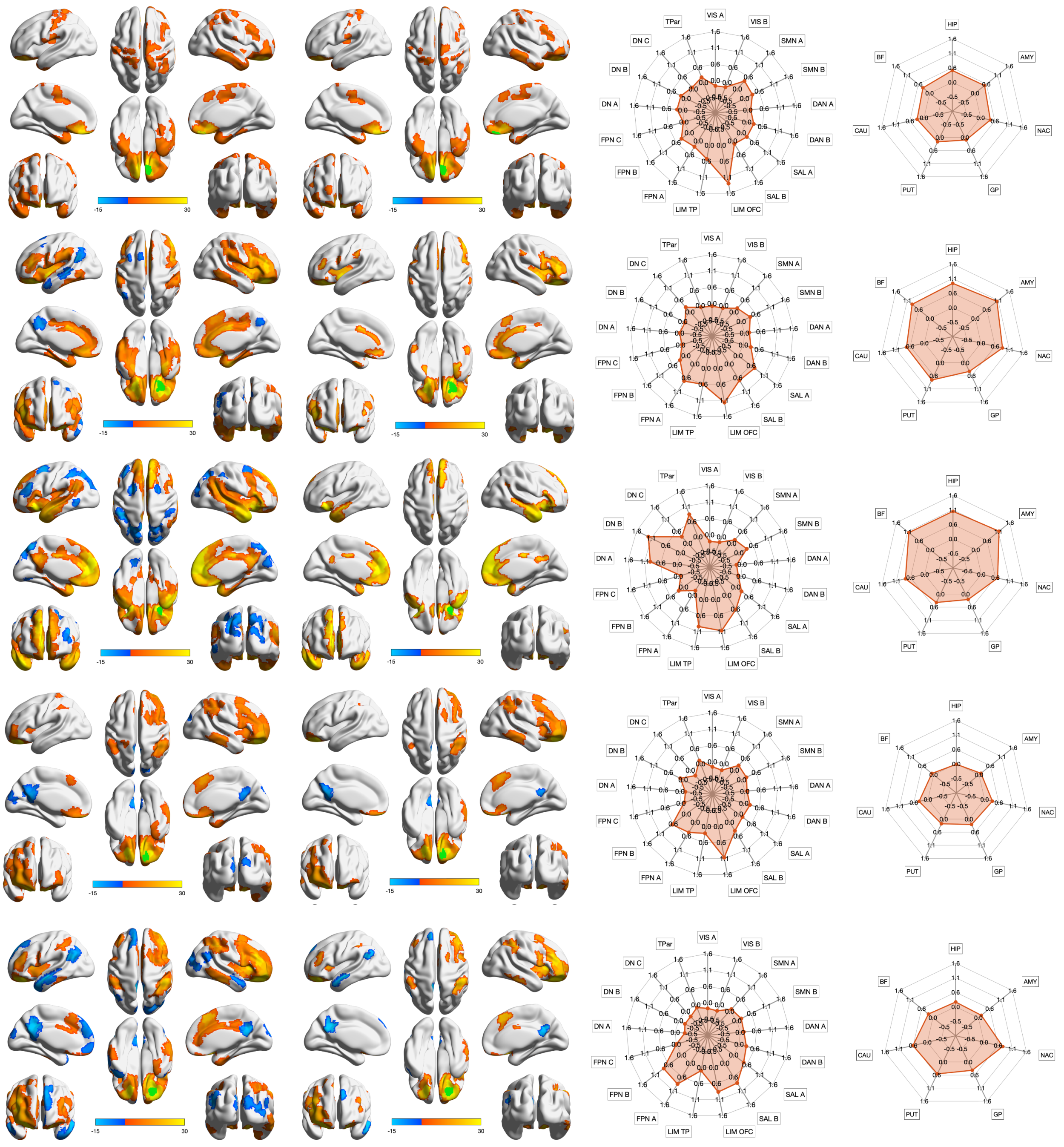

Supplementary Figure 11. Correlation maps for LIM parcels 21-25 (LIM_B), as defined based on the Schaefer-Yeo 1000 region cortical parcellation, averaged across runs. Parcels are shown in light green. Each row corresponds to a given parcel. (Left-Left) The correlation map thresholded at p<0.01, Bonferroni. (Left-Middle) The correlation map thresholded at 90% sparsity. (Right-Middle) Spider plot indicating mean correlation with each of the Yeo et al. (2011) 17-network assignments. (Right-Right) Spider plot indicating mean correlation with each examined subcortical region as per Tian et al. (2020).

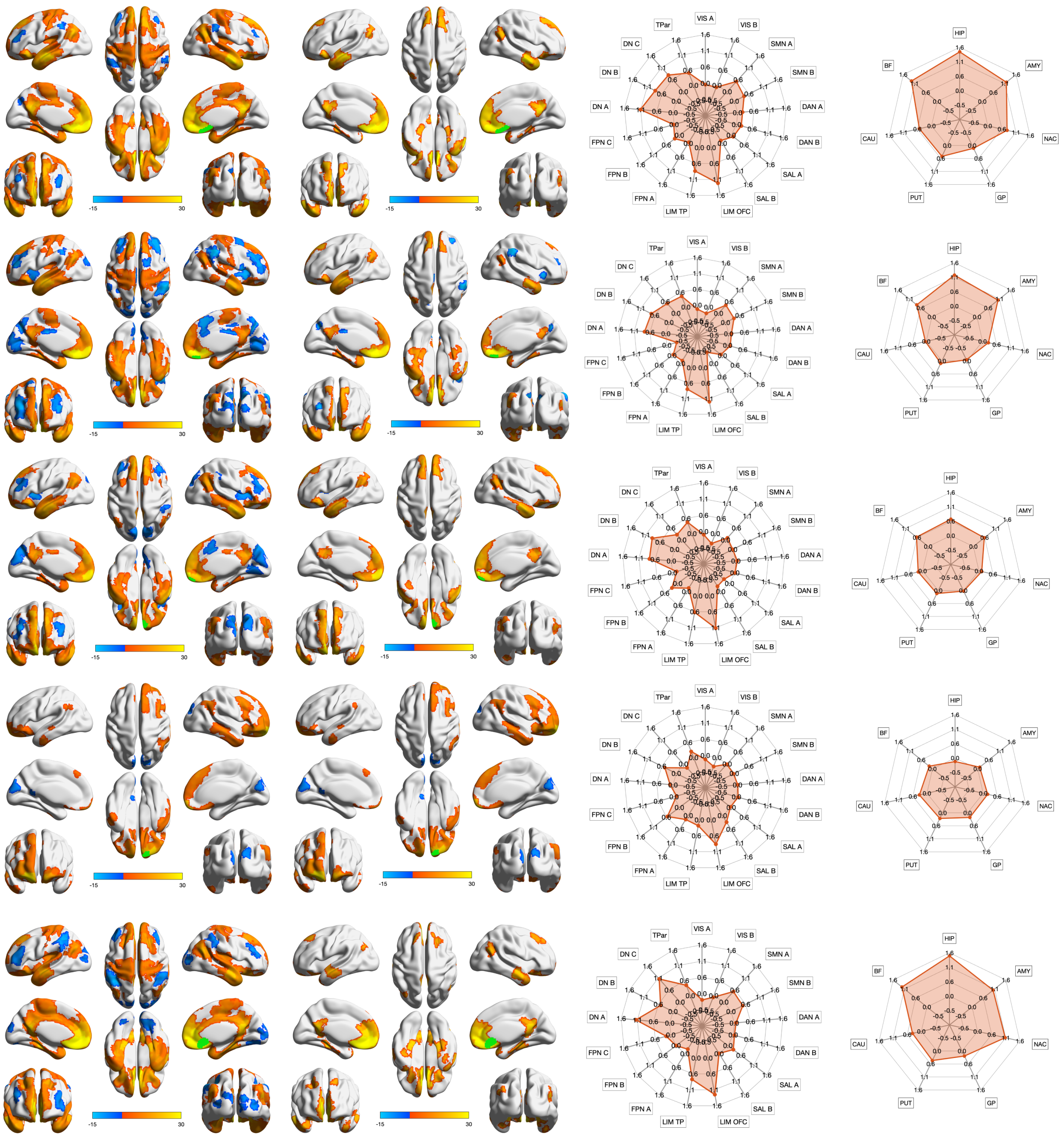

Supplementary Figure 12. Correlation maps for LIM parcels 26-30 (LIM_B), as defined based on the Schaefer-Yeo 1000 region cortical parcellation, averaged across runs. Parcels are shown in light green. Each row corresponds to a given parcel. (Left-Left) The correlation map thresholded at p<0.01, Bonferroni. (Left-Middle) The correlation map thresholded at 90% sparsity. (Right-Middle) Spider plot indicating mean correlation with each of the Yeo et al. (2011) 17-network assignments. (Right-Right) Spider plot indicating mean correlation with each examined subcortical region as per Tian et al. (2020).

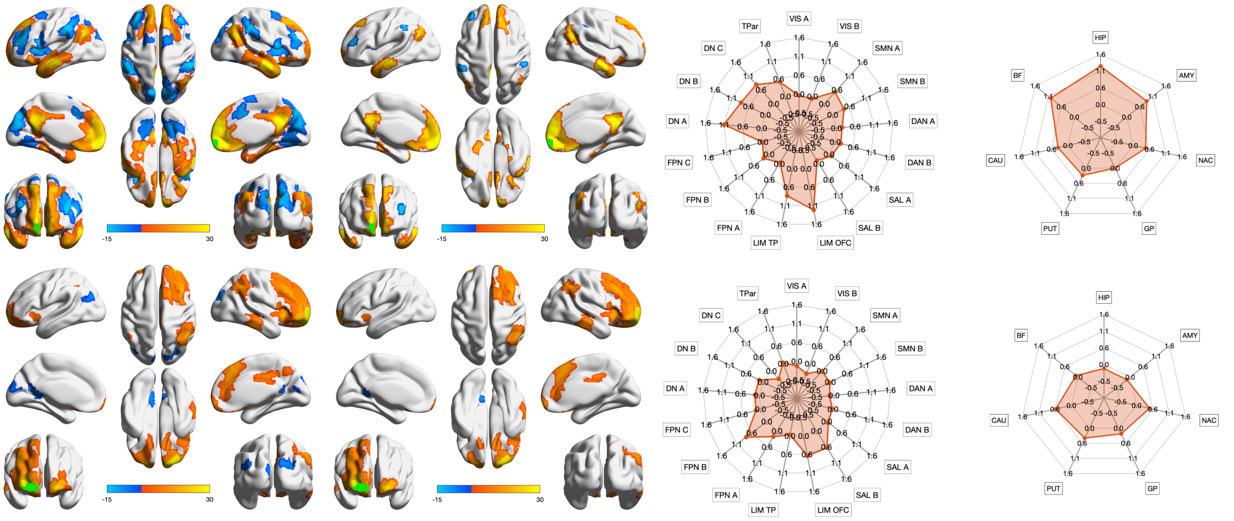

Supplementary Figure 13. Correlation maps for LIM parcels 31-32 ($LIM_{B)}$, as defined based on the Schaefer-Yeo 1000 region cortical parcellation, averaged across runs. Parcels are shown in light green. Each row corresponds to a given parcel. (Left-Left) The correlation map thresholded at $p<0.01$, Bonferroni. (Left-Middle) The correlation map thresholded at 90% sparsity. (Right-Middle) Spider plot indicating mean correlation with each of the Yeo et al. (2011) 17-network assignments. (Right-Right) Spider plot indicating mean correlation with each examined subcortical region as per Tian et al. (2020).

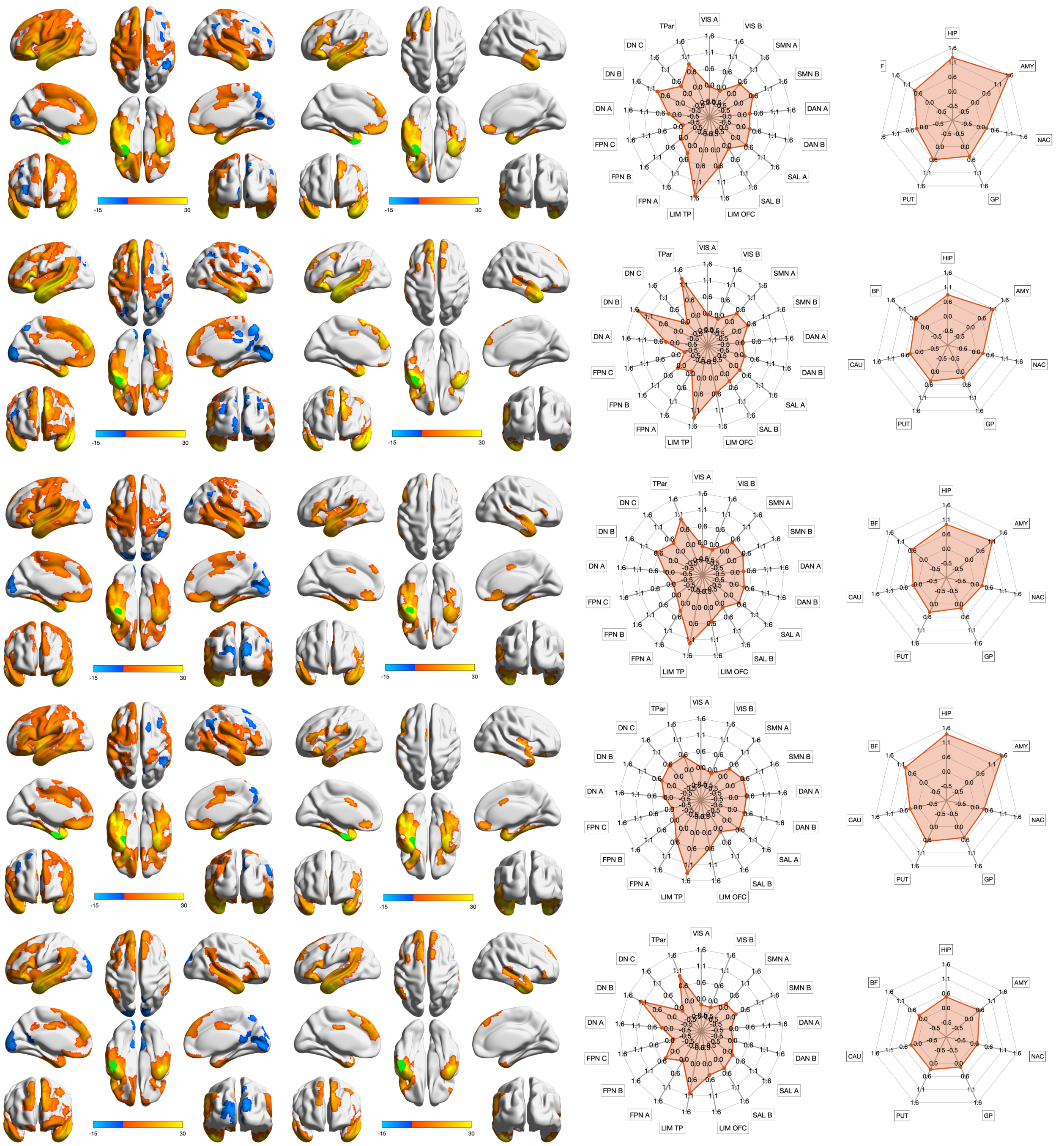

Supplementary Figure 14. Correlation maps for LIM parcels 33-37 (LIM$_A$), as defined based on the Schaefer-Yeo 1000 region cortical parcellation, averaged across runs. Parcels are shown in light green. Each row corresponds to a given parcel. (Left-Left) The correlation map thresholded at p<0.01, Bonferroni. (Left-Middle) The correlation map thresholded at 90% sparsity. (Right-Middle) Spider plot indicating mean correlation with each of the Yeo et al. (2011) 17-network assignments. (Right-Right) Spider plot indicating mean correlation with each examined subcortical region as per Tian et al. (2020).

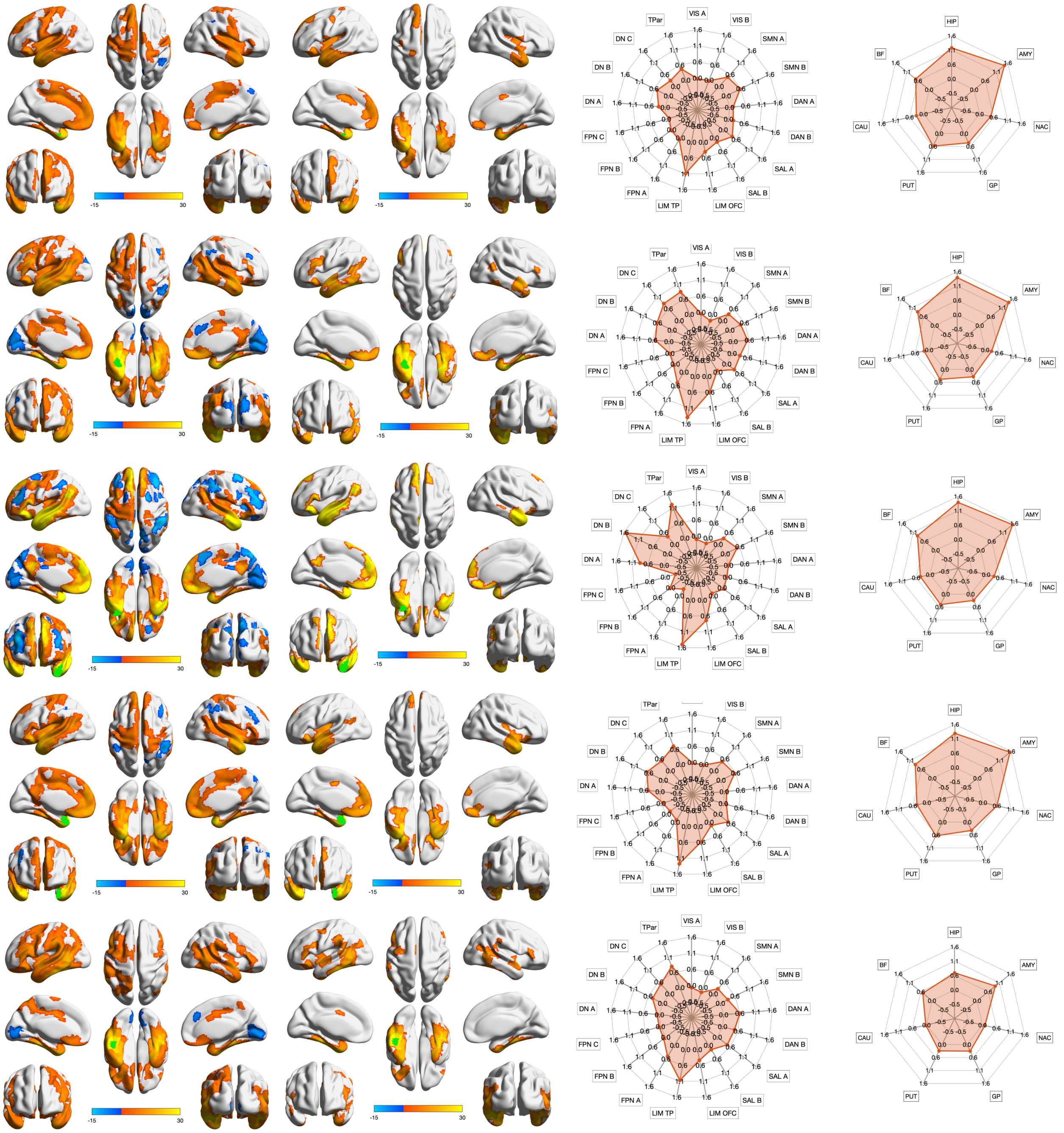

Supplementary Figure 15. Correlation maps for LIM parcels 38-42 (LIM$_A$), as defined based on the Schaefer-Yeo 1000 region cortical parcellation, averaged across runs. Parcels are shown in light green. Each row corresponds to a given parcel. (Left-Left) The correlation map thresholded at p<0.01, Bonferroni. (Left-Middle) The correlation map thresholded at 90% sparsity. (Right-Middle) Spider plot indicating mean correlation with each of the Yeo et al. (2011) 17-network assignments. (Right-Right) Spider plot indicating mean correlation with each examined subcortical region as per Tian et al. (2020).

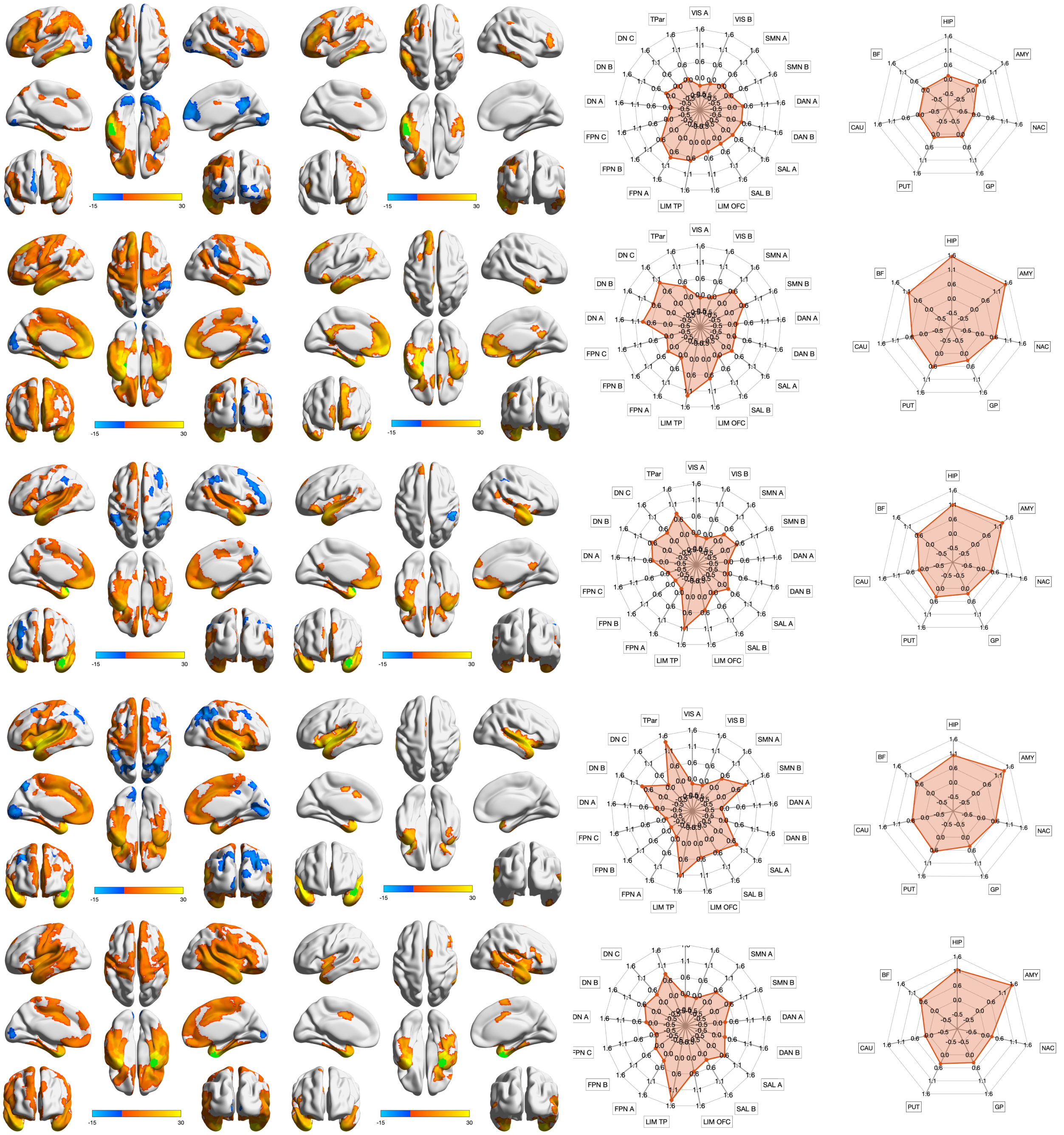

Supplementary Figure 16. Correlation maps for LIM parcels 43-47 (LIM$_A$), as defined based on the Schaefer-Yeo 1000 region cortical parcellation, averaged across runs. Parcels are shown in light green. Each row corresponds to a given parcel. (Left-Left) The correlation map thresholded at p<0.01, Bonferroni. (Left-Middle) The correlation map thresholded at 90% sparsity. (Right-Middle) Spider plot indicating mean correlation with each of the Yeo et al. (2011) 17-network assignments. (Right-Right) Spider plot indicating mean correlation with each examined subcortical region as per Tian et al. (2020).

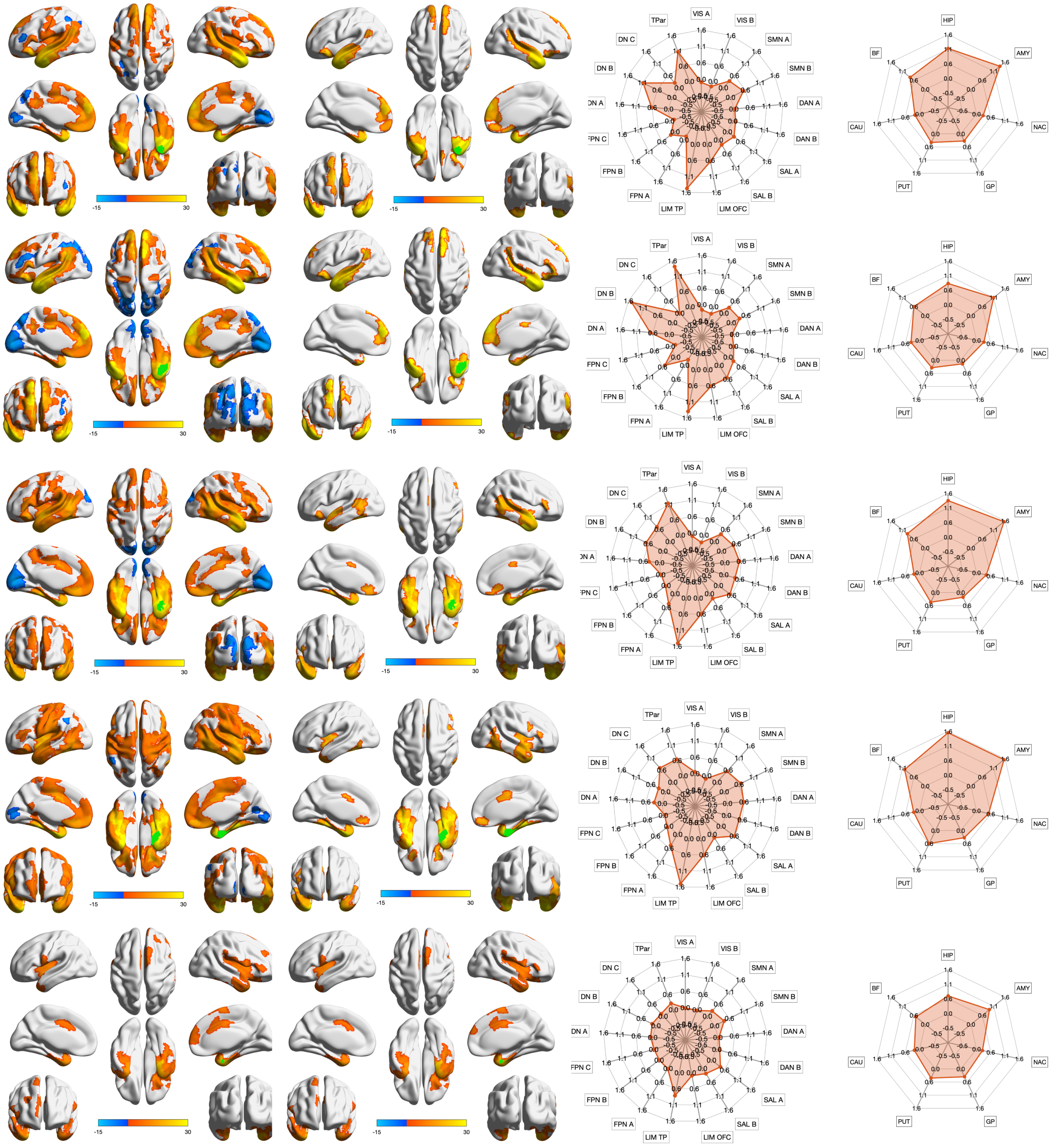

Supplementary Figure 17. Correlation maps for LIM parcels 48-52 (LIM$_A$), as defined based on the Schaefer-Yeo 1000 region cortical parcellation, averaged across runs. Parcels are shown in light green. Each row corresponds to a given parcel. (Left-Left) The correlation map thresholded at p<0.01, Bonferroni. (Left-Middle) The correlation map thresholded at 90% sparsity. (Right-Middle) Spider plot indicating mean correlation with each of the Yeo et al. (2011) 17-network assignments. (Right-Right) Spider plot indicating mean correlation with each examined subcortical region as per Tian et al. (2020).

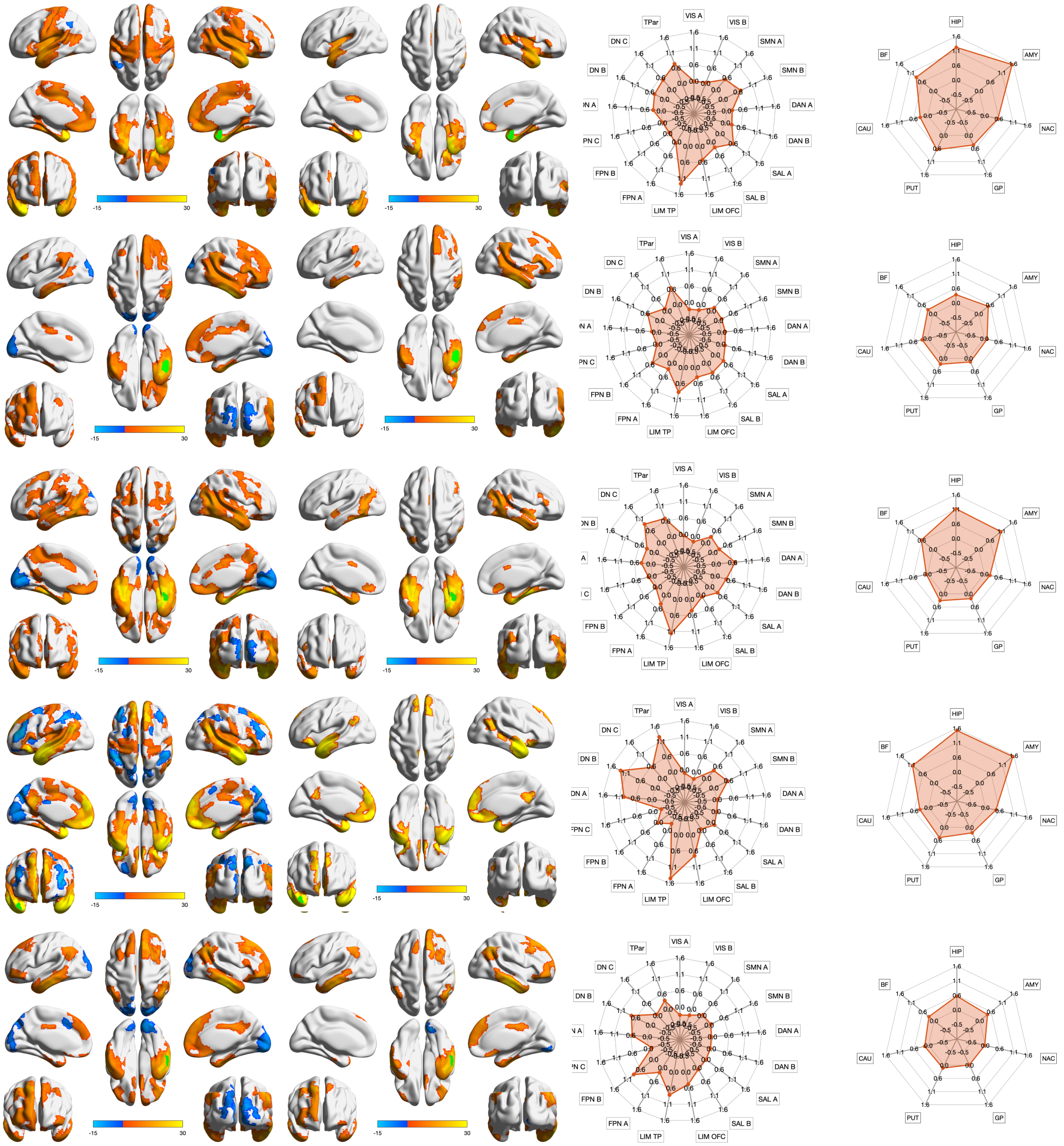

Supplementary Figure 18. Correlation maps for LIM parcels 53-57 (LIM_A), as defined based on the Schaefer-Yeo 1000 region cortical parcellation, averaged across runs. Parcels are shown in light green. Each row corresponds to a given parcel. (Left-Left) The correlation map thresholded at $p<0.01$, Bonferroni. (Left-Middle) The correlation map thresholded at 90% sparsity. (Right-Middle) Spider plot indicating mean correlation with each of the Yeo et al. (2011) 17-network assignments. (Right-Right) Spider plot indicating mean correlation with each examined subcortical region as per Tian et al. (2020).

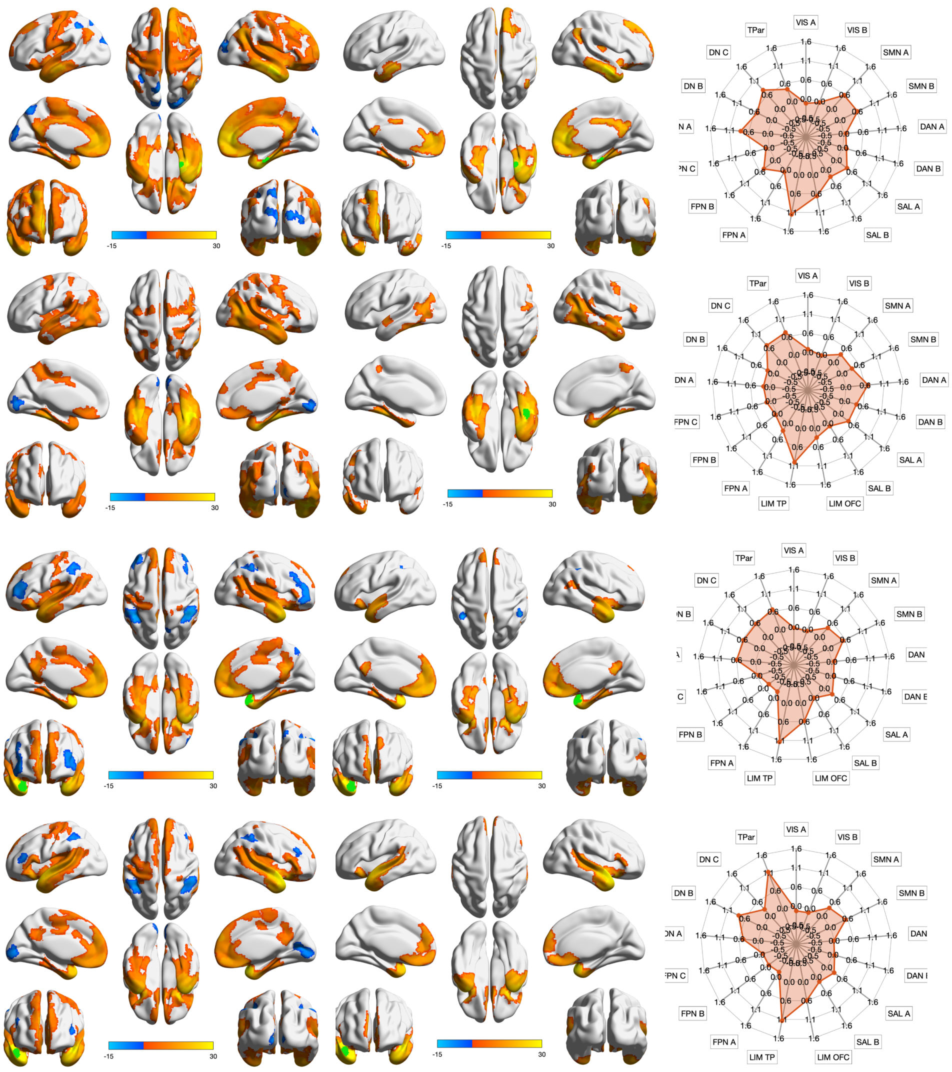

Supplementary Figure 19. Correlation maps for LIM parcels 58-62 (LIM_A), as defined based on the Schaefer-Yeo 1000 region cortical parcellation, averaged across runs. Parcels are shown in light green. Each row corresponds to a given parcel. (Left-Left) The correlation map thresholded at p<0.01, Bonferroni. (Left-Middle) The correlation map thresholded at 90% sparsity. (Right-Middle) Spider plot indicating mean correlation with each of the Yeo et al. (2011) 17-network assignments. (Right-Right) Spider plot indicating mean correlation with each examined subcortical region as per Tian et al. (2020).
